# A DNA methylation map of human cancer at single base-pair resolution

**DOI:** 10.1038/onc.2017.176

**Published:** 2017-06-05

**Authors:** E Vidal, S Sayols, S Moran, A Guillaumet-Adkins, M P Schroeder, R Royo, M Orozco, M Gut, I Gut, N Lopez-Bigas, H Heyn, M Esteller

**Affiliations:** 1 Cancer Epigenetics and Biology Program (PEBC), Bellvitge Biomedical Research Institute (IDIBELL), Barcelona, Spain; 2 Centre for Genomic Regulation (CRG), Barcelona Institute of Science and Technology (BIST), Barcelona, Spain; 3 Universitat Pompeu Fabra (UPF), Barcelona, Spain; 4 CNAG-CRG, Centre for Genomic Regulation (CRG), Barcelona Institute of Science and Technology (BIST), Barcelona, Spain; 5 Biomedical Genomics Lab, Research Program on Biomedical Informatics, IMIM Hospital del Mar Medical Research Institute and Universitat Pompeu Fabra, Barcelona, Spain; 6 Joint IRB-BSC Program in Computational Biology, Institute for Research in Biomedicine (IRB Barcelona), The Barcelona Institute of Science and Technology, Barcelona, Spain; 7 Institute for Research in Biomedicine (IRB Barcelona), Barcelona, Spain; 8 Department of Biochemistry and Molecular Biology, University of Barcelona, Barcelona, Spain; 9 Institució Catalana de Recerca i Estudis Avançats (ICREA), Barcelona, Spain; 10 Department of Physiological Sciences II, School of Medicine, University of Barcelona, Barcelona, Spain

## Abstract

Although single base-pair resolution DNA methylation landscapes for embryonic and different somatic cell types provided important insights into epigenetic dynamics and cell-type specificity, such comprehensive profiling is incomplete across human cancer types. This prompted us to perform genome-wide DNA methylation profiling of 22 samples derived from normal tissues and associated neoplasms, including primary tumors and cancer cell lines. Unlike their invariant normal counterparts, cancer samples exhibited highly variable CpG methylation levels in a large proportion of the genome, involving progressive changes during tumor evolution. The whole-genome sequencing results from selected samples were replicated in a large cohort of 1112 primary tumors of various cancer types using genome-scale DNA methylation analysis. Specifically, we determined DNA hypermethylation of promoters and enhancers regulating tumor-suppressor genes, with potential cancer-driving effects. DNA hypermethylation events showed evidence of positive selection, mutual exclusivity and tissue specificity, suggesting their active participation in neoplastic transformation. Our data highlight the extensive changes in DNA methylation that occur in cancer onset, progression and dissemination.

## Introduction

Over the last decade, genetic research has moved on from the use of targeted approaches to the routine application of exome and genome sequencing. The wealth of genomic data has yielded profound insights into human variation and disease biology. However, screening the genome at base-pair resolution has also shown that the naked genetic code alone is not sufficient to explain the complexity of life. Hence, focus has shifted toward gene regulation and the mechanisms controlling gene expression and post-transcriptional activity.^1^ DNA methylation participates in this closely connected regulatory network by covalently modifying the genetic code, thereby forming the epigenetic code.^2^

Despite an identical genomic blueprint, cells develop into phenotypically distinct cell types to form the human organism. In somatic tissues, the DNA methylation profile defines tissue identity^3^ and is largely conserved over a lifetime, although specific changes occur as life progresses.^4^ In addition, external stimuli, such as lifestyle and environment, are capable of introducing DNA methylation alterations associated with phenotypic changes, including disease susceptibility.^5^ Accordingly, aberrant DNA methylation has been reported for diverse diseases, with cancer being associated with the most profound changes.^6^ Specific DNA methylation changes and genome-wide alterations are involved in all steps of tumorigenesis.^7^ In particular, the epigenetic silencing of tumor-suppressor genes (TSGs) is generally thought to be of great importance to cancer onset and to driving tumorigenesis.

However, to date, technical restrictions have meant that the extent of DNA methylation alterations in human cancers could only be estimated. For this reason, comprehensive whole-genome efforts are required to evaluate the complexity of DNA methylation landscapes in tumors and their contribution to oncogenesis. Accordingly, in this study, we comprehensively profiled the DNA methylation landscape of >1000 normal and cancer samples genome wide or at genome scale. Here, landscape features obtained from few comprehensively profiled samples, including cancer cell lines, could be replicated in large primary tumor cohorts and genome-scale technologies, narrowing candidate regions down to specific cancer-related aberrations. We recently showed the value of genome-wide DNA methylation profiling for the identification of functional variance at super-enhancer regions.^8^ Here, we extend this work by enlarging the number of cancer types and performing an unbiased interpretation in a data-driven manner. This work expands previously reported findings by cancer-specific studies^3, 9, 10, 11, 12, 13, 14, 15, 16, 17^ or The Cancer Genome Atlas (TCGA) efforts and extends these studies by the genome-wide profiling for putative epigenetic cancer driver events across a broad spectrum of cancer types.

## Results

### Genome-wide changes of DNA methylation in cancer

In order to gain a comprehensive insight into the variation in DNA methylation between normal tissue types and alterations occurring in different cancer contexts, we performed genome-wide profiling of 22 human samples using whole-genome bisulfite sequencing (WGBS). WGBS creates DNA methylation profiles at base-pair resolution to give an unbiased overview of the DNA methylation landscape. The analyzed samples included normal tissue types and their associated neoplasms, which are among the most abundant cancer types worldwide. In particular, we used WGBS with eight normal tissue types and 13 associated cancer samples (Table 1). Thirteen of these cases were publicly available from our in-house WGBS pipeline.^8^ Normal samples included brain, blood, breast, prostate, liver, lung, colon and placenta specimens. Brain tissue was represented by matched white and gray matter frontal cortex samples to account for different cell-type compositions. To analyze DNA methylation variation from different perspectives, we produced reference data sets for cancer samples that included primary tumors and cancer cell lines. Changes during tumor progression were analyzed using matched primary and metastatic samples for breast and colorectal cancer specimens. The latter in particular were a source of highly valuable information, as the sequenced triplet (normal, primary cancer and liver metastasis) was donor matched.

**Figure 1:**
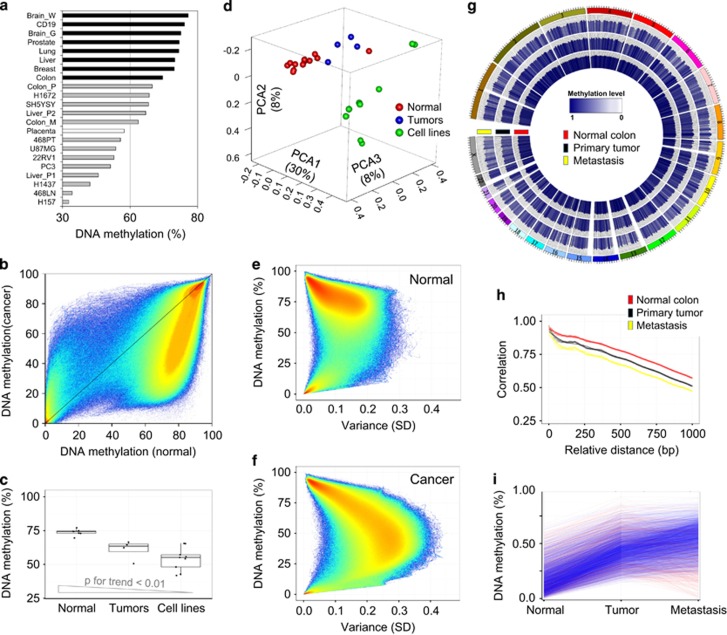
Genome-wide changes in DNA methylation in human cancer. (**a**) Genome-wide DNA methylation levels of 22 human samples determined by whole-genome shotgun bisulfite sequencing (WGSBS). Samples are ordered by DNA methylation values and normal (black), cancer (gray) and placenta (white) samples are color coded. (**b**) Average DNA methylation levels of normal versus cancer samples. Each dot represents the average DNA methylation level at a single CpG site in normal (*x* axis) or cancer (*y* axis) samples. (**c**) Total DNA methylation levels of normal tissues, tumor samples and cancer cell lines. (**d**) Principal component analysis (PCA) of DNA methylation levels using WGBS data from 22 human samples. (**e**, **f**) Average CpG methylation levels and standard variation (s.d.) of normal tissue types (**e**) and cancer samples (**f**). (**g**) Whole-genome representation of DNA methylation levels (5 Mbp windows) of donor-matched normal colon, primary colorectal cancer and liver metastasis samples. (**h**) Correlation of DNA methylation levels between neighboring CpG sites in normal colon, primary colorectal cancer and liver metastasis samples. (**i**) Average DNA methylation levels of hypermethylated regions comparing normal colon with the matched colorectal tumor. Consistent DNA hypermethylation in the metastasis sample is indicated in blue (red, otherwise).

Bisulfite sequencing reads (median ~600 million per sample) were mapped to the references genome (HG19) using the BISMARK algorithm, which gave a median coverage of 13x per sample (range 6 × to 25 ×, Table 1). Consistent with a previous report,^3^ normal tissue samples had an average CpG methylation level of 70–80%, whereas cancer samples displayed severe losses in this context (Figures 1a and b, Table 1). Global DNA methylation was significantly lower in primary cancer samples compared with normal tissues, and even lower in cancer cell lines (*P*<0.01; Figure 1c). Cancer cell lines were previously described to harbor specific epigenetic peculiarities,^3, 4, 5, 6, 7, 8, 9, 10, 11^ likely introduced by culture and selection processes, and consequently our WGBS-derived findings were extensively validated using larger cohorts of primary cancer samples.

It is of note that the 58% global CpG methylation level in placenta tissue differed markedly from that in other normal tissue types (Figure 1a), this result being in line with a previous studies reporting the existence of large partially methylated domains in this tissue type.^18, 19^ Principal component analysis indicated that 46% of the total variance was explained by the first three principal components, which clearly separated normal from cancer samples and primary tumors from cancer cell lines (Figure 1d). Whereas normal samples clustered close together, cancer samples were highly variable even within the same cancer types (Supplementary Figure S1). Consistently, we observed less variance at single CpG sites among normal tissue types (34%, s.d.>0.1, Figure 1e) than in cancer samples (64.8%, s.d.>0.1, Figure 1f).^20, 21^ In line with previous studies, we detected a significant decrease in DNA methylation levels in the majority of repetitive elements (RepeatMasker, Student’s *t*-test, *P*<0.01; Supplementary Figure S2).

We found a progressive decrease in global DNA methylation from healthy tissues to the primary tumors and, in turn, to their associated metastases, suggesting a successive loss of DNA methylation during tumorigenesis (Figure 1g; Supplementary Figure S3a). This phenomenon was further underlined by principal component analysis, which revealed increasing distances as cancer progressed (Supplementary Figure S1). The decrease in the level of DNA methylation resulted in a more heterogeneously methylated genome, illustrated by the loss of the association between methylation levels of neighboring CpG sites, indicating that hypomethylation occurred randomly rather than at distinct consecutive CpG sites (Figure 1h; Supplementary Figure S3b). The oncogenic progression in the colon triplet could also be observed at loci that gain DNA methylation, such as differentially methylated regions hypermethylated in the primary tumor compared with the matched healthy tissue, which further gained methylation intensities in the metastasis (Figure 1i). Despite the increase in DNA methylation intensity, the number of hypermethylated differentially methylated regions increased only marginally, from 12 364 to 15 373, between the samples taken from the primary colon tumor or the matched liver metastases. Considering that the number of hypomethylated differentially methylated regions more than doubled from the primary colon tumor to the metastasis (87 663–205 459), resulting in global DNA hypomethylation, we hypothesize that DNA methylation is progressively lost during tumorigenesis, whereas DNA hypermethylation may underlie positive clonal selection in the metastatic process.

### DNA methylation-based segmentation of the human genome

Functional alterations of DNA methylation in human cancer overlap with regulatory important regions, such as the transcription start site or enhancers. Given the impact of DNA methylation on transcriptional activity and hence cellular phenotypes, we aimed to comprehensively profile the DNA methylation landscape for regions actively contributing to gene regulation in normal tissues and to determine their variation among cancers. Although genome wide, the majority of CpGs are highly methylated, distinct regions display strikingly lower methylation levels at consecutive CpG sites. These hypomethylated regions (HMRs) are associated with the accessibility of the transcriptional machinery and transcription factors, and mark epigenetically active sites within the DNA methylome.^15, 22, 23, 24, 25^ Promoters of transcribed genes consistently harbor HMRs, and beyond the promoter context HMRs are believed to mark *cis*-regulatory elements. Hence, we suggest that the DNA methylation landscape is an informative genomic feature that facilitates the interpretation of the genetic code and the identification of functional alterations in human cancers.

We assembled a comprehensive HMR catalog using our WGBS data sets, creating a genome-wide map of loci active in regulation. In total, we identified 116 628 unique HMRs in normal tissue samples, ranging from 43 259 to 66 711 in lung and prostate, respectively (Table 1). With a geometric mean of 830 bp, HMRs covered 2% of the entire genome. In all, 31.1% (36 229) of the HMRs were located within gene promoters (GENCODE v16.0, Hinxton, Cambridgeshire, UK), 36.4% (42 398) were intragenic and 32.8% (38 001) were located outside of the transcriptional context. The relationship of HMRs to active regulatory sites was underlined by the fact that ⩾95% of active and poised promoters and 60% of strong enhancers overlapped with focal hypomethylated loci in breast tissue with similar trends were seen in blood and liver cell types (Supplementary Figure S4).

**Figure 2:**
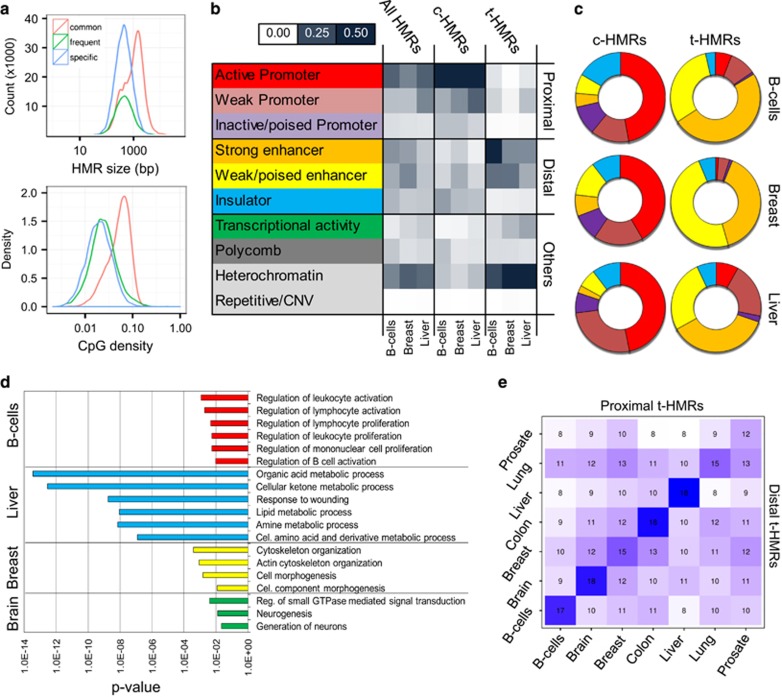
Functional characterization of HMR subtypes. (**a**) Size and CpG density distribution of common, frequent and t-HMRs in normal B cells. (**b**) Localization of HMRs defined for B cells, breast and liver tissues within histone mark defined segments of the genome. The distribution is displayed as overlap frequencies. (**c**) Distribution of common (c-HMRs) and t-HMRs within regulatory regions defined for normal B cells, breast and liver tissues. (**d**) Enrichment analysis in biological processes for gene promoters harboring t-HMRs (Bonferroni adjusted *P*-value). (**e**) Co-occurrence frequency of promoter-associated (proximal) and distal t-HMRs between normal tissue types.

Surprisingly, only 23.0% of the HMRs (26 855) were common for all cell types (c-HMR) and additional 25.7% (29 960) were frequently observed (>50% samples). In total, 57.8% (15 510) of c-HMRs colocalized with gene promoters, including human housekeeping genes, such as GAPDH, TBP and PPIA (Supplementary Figure S5). Conversely, 46.4% (54 134) of HMRs were tissue-specific HMR (t-HMR) with B cells presenting the highest (14 092) and lung (1226) the lowest number (Supplementary Table S1). The t-HMRs were of smaller size and presented less CpG density compared with their common counterparts (Figure 2a). By integrating histone modifications data, enabling a genome-wide categorization of regulatory regions and activity states,^26^ we observed that c-HMRs were mainly located in gene promoters, whereas t-HMRs were frequently present at distal *cis*-regulatory enhancers regions (Figures 2b and c). The unequal distribution between c- and t-HMRs suggests that the epigenetic identity of distinct tissues mainly to be defined outside the promoter context and specifically at hypomethylated distal enhancer sites, which is in line with previous studies analyzing different normal tissue and cell types.

Functionally, t-HMRs in gene promoters were significantly enriched in genes presenting tissue-specific expression in the respective tissue types (TiGER, Fisher’s test, *P*<0.01). Also, we detected a compelling enrichment of t-HMR-related genes in biological processes directly associates to their respective cell-type and function. For examples, blood, liver and brain-specific promoter-associated t-HMRs were enriched for leukocyte activation, organic acid metabolic processes and neurogenesis, respectively (Bonferroni adjusted *P*<0.05, Figure 2d). Importantly, functional enrichment was not only identified for t-HMRs located in promoters, but also for those present outside (<10kb from TSS) the direct regulatory context and supporting the importance of proximal and distal t-HMRs for tissue-specific functions (Supplementary Figure S6). We further reasoned that distal t-HMRs are associated with active genes in their proximity. Accordingly, we determined the degree of co-activation between t-HMRs and proximal genes within the different tissue types, considering hypomethylated promoters as potentially active. Consistent with their suspected function t-HMR revealed co-activation of neighboring genes more likely within their respective tissue context than within unrelated types (Figure 2e).

In order to confirm the presence of c- and t-HMRs, we analyzed a larger set of normal tissue types on the HumanMethylation450 BeadChip, an array-based DNA methylation platform interrogating more than 450 000 CpG sites.^27^ Herein, the BeadChip analyzes DNA methylation levels of 99% of gene promoters and additional intra- and intergenic regulatory loci. Technically, 38.2% (44 547) of WGBS-derived HMRs from normal tissues were represented on the array platform. We integrated DNA methylation profiles from 114 normal specimens, representing the entire set of tissue types in the analysis. Importantly, >95% of c-HMRs were confirmed to be hypomethylated throughout all tissue types (>5 probes, average DNA methylation ≤30%, Supplementary Figure S7a). Also t-HMRs could be identified with a sensitivity of up to 85% (false positive rate <10%, >2 probes; area under the curve=0.95), however, with strong differences between tissue types (Supplementary Figure S7b). In particular, only 33% and 20% of breast and prostate specific HMRs could be validated (false positive rate <10%), respectively, suggesting a high degree of inter-individual variation and possibly related to the hormone-responsive nature the tissues. Nevertheless, hierarchical clustering of the 9391 t-HMRs interrogated by the BeadChip were capable the separate the analyzed normal samples in respect to their tissue of origin (Supplementary Figure S8).

### Chasing functional DNA hypermethylation events in cancer

**Figure 3:**
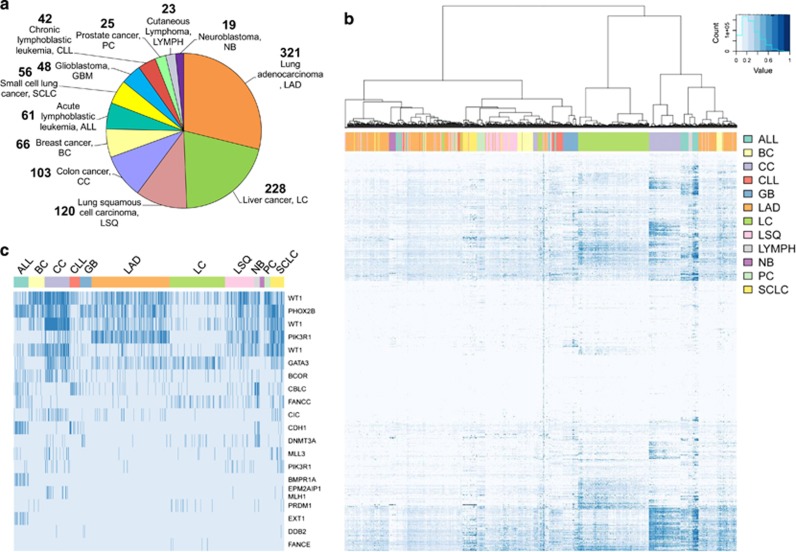
Genome-scale DNA methylation analysis of 1112 cancer samples reveals cancer-type-specific signatures. (**a**) Cancer samples analyzed on HumanMethylation450 BeadChip. (**b**) Unsupervised hierarchical clustering of 1112 cancer samples and 8254 hypermethylated c-HMRs within gene promoters using Jaccard distances and Ward cluster method. Average HMR methylation levels are continuously color-coded from 0% (light blue) to 100% (dark blue). (**c**) Frequently hypermethylated HMRs in TSG promoters in 1112 cancer samples ordered by type. Average HMR methylation status is categorized as hypermethylated (>33% DNA methylation, dark blue) and hypomethylated (<33% DNA methylation, light blue).

In an attempt to comprehensively describe the variability among human cancer HMRs, we further interrogated DNA methylation at the genome scale in 1112 primary samples using the HumanMethylation450 BeadChip. This set of primary samples represented cancer types directly related to previously interrogated tissue types. Overall, we analyzed 12 tumor types, with a median representation of 59 samples (ranging from 19 to 321, Figure 3a). In order to detect cancer-specific variance in this sample cohort, we focused on c-HMR considering their potential role in tissue maintenance and homeostasis. From the 19 686 c-HMRs that were consistently hypomethylated in normal tissues, 11.6% (2279) exhibited frequent DNA hypermethylation events in the 1112 cancer specimens (average DNA methylation >33% in at least 25% of the samples) (Supplementary Figure S9a). Analyzing similarities within promoter-associated c-HMRs between tumor samples revealed that cancer samples clustered with respect to their tissues of origin, suggesting the existence of specific cancer-type DNA methylation signatures (Figure 3b). This was equally true for c-HMRs outside the promoter context (Supplementary Figure S10a), where there was an even higher variance in cancer samples compared with promoter-related HMRs (Supplementary Figure S9b). However, we also detected substantial differences within the cancer types, whereby the subgroups were clearly separated by the cluster approach. These findings were in line with the previous observation that subsets of tumor samples have elevated DNA hypermethylation frequencies.

In regard to their genomic location and putative functional impact, we observed DNA methylation gains at c-HMRs mainly occurred at poised promoters and repressed chromatin sites, being a consistent phenomenon across cancer types (Supplementary Figure S11). In line, the 100 most frequently hypermethylated genes were significantly enriched in developmental processes (gene ontology, biological processes, false discovery rate<0.01) and homeobox structures (INTERPRO, false discovery rate<0.01) (Supplementary Table S2), which is consistent with previous cancer studies that reported a gain of DNA methylation in genes related to development and differentiation, such as Polycomb target genes. Hypermethylation events in developmental genes are thought to promote a more undifferentiated cell state by stably blocking genes that regulate the transition from stem cell configuration to somatic differentiation.^28^ As these events are likely to be involved in the formation of a tumor-promoting environment, they are considered to support rather than to drive cells into uncontrolled growth.

To identify the functional epigenetic events that actively contribute to cancer cell transformation, we interrogated the data set using strategies specifically tailored to epigenetic data mining. In particular, we assumed that functional DNA methylation events follow similar rules to those of cancer-driving genetic alterations: (1) known TSGs are silenced by promoter hypermethylation; (2) functional hypermethylation events of TSG can be mutually exclusive and (3) enriched in a cancer-type-specific manner.

We screened genes previously reported as being cancer genes (COSMIC Cancer Gene census) for variation in promoter-associated c-HMRs (rule 1). Consistent with their suspected guardian function, protecting cells from oncogenic transformation, we detected several TSG promoters that were hypermethylated in human cancer samples (Figure 3c). Although genes like *WT1*, *GATA3* and *PHOX2B* were frequently hypermethylated in several cancer types, others were restricted to certain tissue types. Specifically, promoter hypermethylation of *PIK3R1*, a repressor of the mitogenic AKT pathway,^29^ was mainly restricted to lung cancer subtypes. *PIK3R1* is frequently mutated in lung cancer and its hypermethylation was significantly associated with gene repression in primary lung cancer samples (TCGA, LUAD, Pearson’s correlation, rho=–0.29, *P*<0.01). Taking advantage of our comprehensive segmentation of the DNA methylation landscape, we further investigated c-HMR outside the promoter context (<50-kb distant from TSS). Our examination of these distal regulatory loci enabled us to identify additional DNA hypermethylation events in c-HMRs near TSGs, such as *FANCC*, *CYLD*, *MSH2*, *PRDM1*, *ARID1A* and *BCOR* (Supplementary Figure S10b). The gain of DNA methylation near the DNA repair gene *MSH2* (–31 972 bp) was almost exclusively restricted to, and highly frequent in, liver cancer specimens. However, other variations were present in a broader spectrum of affected tissue types.

In a second approach to identify functional epigenetic events (rule 2), we analyzed the mutual exclusivity of the DNA hypermethylation events of putative TSGs that are frequently altered by mutations,^30^ deletions^31^ or hypermethylation.^32^ To avoid tissue-related biases, we analyzed hypermethylated gene promoters separately for each cancer type. Genes involved in non small cell lung cancer pathogenesis (KEGG ID: hsa05223) were mutually exclusive in lung carcinomas (Supplementary Figure S12a). In particular, hypermethylation of *PIK3R5*, *CDKN2A* and two retinoid X receptors (*RXRG* and *RXRG*) occurred in a non-redundant manner in the non small cell lung cancer subtypes (Z score=2.82, empirical *P*<0.01). We also noticed that hypermethylation of phosphoinositide-3-kinase subunits was mutually exclusive in all three lung cancer subtypes (Z score=4.14, empirical *P*<0.01, Supplementary Figure S12b), suggesting a disease-driving effect of the single alterations within the apoptosis signaling cascade in lung cancer. As lung cancer susceptibility is closely related to the smoking status of patients, we were interested to assess the involvement of epigenetic alteration in detoxifying enzymes in disease biology. We observed mutually exclusive hypermethylation of genes involved in chemical carcinogenesis (KEGG ID: hsa05204). In particular, frequent alteration in *GSTT1*, *ALDH3A3, CBR1* and *GSTM3* showed non-redundant events in the three lung cancer subtypes (Z score=2.00, empirical *P*<0.05, Supplementary Figure S12c). Hence, we suggest epigenetic alteration displays similar characteristics to genetic aberration and that mutual exclusivity analysis is a powerful method for identifying functional epigenetic events in cancer.

In another attempt to identify novel cancer-driving events (rule 3), we performed enrichment analysis of DNA hypermethylation events in putative TSGs^30, 31, 32^ for the cancer types, assuming that tissue-specific events have a role in the oncogenic transformation of their respective tissue types. We were able to validate previous findings describing frequent hypermethylation events of *EPHA7*, *MGMT* and *MLH1* in colorectal cancer patients, *RARB* in prostate tumors, and *CASP8* in glioblastoma (Supplementary Figure S13). We identified additional, yet poorly recognized relationships between epigenetic silencing and tissue-specific carcinogenesis. For example, colorectal patients harbored recurrent hypermethylation of extracellular matrix components of the TSGs CDM2, COL18A1 and MMP2,^33^ and lymphoma patients frequently exhibited elevated promoter methylation levels of *CAST*, *NCOA4*, *LYN* and *DNMT3A*. Interestingly, *DNMT3A* is frequently mutated in acute myeloid leukemia patients and so its epigenetic silencing in lymphoma patients could also drive tumorigenesis in this context.^34^ In addition, as LYN functions as a B-cell receptor regulator, its repression could directly participate in altered B-cell behavior and tumor formation.^35^

Finally, we hypothesized that owing to the selection process during tumorigenesis resulting in an outgrowth of tumor sub-clones, important functional epigenetic changes can be identified by assuming an overrepresentation of positively selected signals within the tumor mass. Consequently, putative epigenetic driver events could present elevated DNA methylation intensities, reflecting the greater abundance of cells harboring the respective alterations. Therefore, we selected c-HMRs containing gene promoters with recurrent (>1% frequency) promoter hypermethylation at high intensities (average HMR methylation level >50%) and represented by at least two detection probes on the BeadChip. Further, we focused on solid tumor types, as clonal selection in leukemia potentially follows different dynamics. This alternative approach highlighted potential epigenetic driver-gene candidates that were highly depleted in developmental genes, underlining the value of this identification strategy. In total, we identified 231 HMRs with elevated DNA methylation levels in cancer (average HMR methylation level >50%). Candidate genes included several known TSGs (Supplementary Table S3), including *MLH1*, which is frequently hypermethylated in colorectal cancer. Also, *CDKN2A* and *XRCC5*, which are TSGs involved in DNA repair, displayed high hypermethylation intensities and, unlike *MLH1*, were altered in all tumor types. Other HMRs exhibiting substantial DNA methylation gains included genes involved in cell motility (*DCDC2*), senescence (*SRF*) and differentiation (*ADHFE1*), as well as the colorectal biomarker *SEPT9*.

### The form and function of cancer DNA methylomes

**Figure 4:**
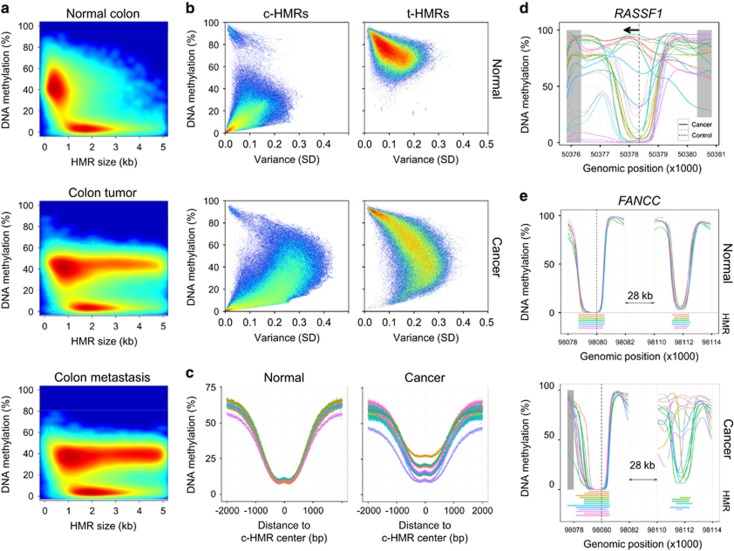
HMR subtypes are highly variable in human cancer samples. (**a**) Size distribution and DNA methylation levels of total HMRs in donor-matched normal colon, primary cancer and metastasis samples. Densities are displayed as low (blue) to high (red). (**b**) Average DNA methylation levels and standard variation (s.d.) of common (c-HMRs) and t-HMRs in normal tissue (upper panel) and cancer samples (lower panel). (**c**) DNA methylation structure of c-HMRs in normal and cancer samples. CpG methylation levels are averaged in 4-bp windows over all c-HMRs and displayed ±2-kb flanking the HMR center. (**d**) DNA methylation profiles of the *RASSF1* promoter in normal (broken lines) and cancer samples (solid lines). (**e**) DNA methylation profiles of the *FANCC* promoter and a distal c-HMR in normal (upper panel) and cancer samples (lower panel).

Following the interpretation of specific functional regions within the genome, we extended the analysis to the genome-wide characterization of cancer-related alterations of the DNA methylation landscape. Consistent with previous studies of single cancer types,^10, 11^ we observed the formation of large intermediately methylated blocks in all the cancer samples analyzed, which defines an epigenetic hallmark of human cancers (Figure 4a; Supplementary Figure S14). These regions were previously described as partially methylated domains and likely display distinct effects on genome regulation compared with HMRs, unmethylated regions or lowly methylated regions (Supplementary Figure S15).^24, 36^ There were large differences between the samples even within cancer types, whereby HMRs covered only 16.9% of all base pairs in the small cell lung cancer genome, but 38.2% of base pairs in the lung squamous cell carcinoma sample (Table 1). From the regulatory point of view, cancer-specific HMRs (>1,500 bp) were depleted in HMRs as determined in a matched healthy context (Fisher’s exact test, *P*<0.01), implying that global loss of DNA methylation in cancer occurs in regions of weak regulatory activity, such as highly methylated sites in normal tissues and being in line with previous observations in partially methylated domains in cancer.^9^ This was further supported by the fact that HMRs formed *de novo* were enriched in intergenic regions compared with transcribed sequences, and in intronic loci compared with exons (Fisher’s exact test, *P*<0.01). It is of note that we detected progressive DNA methylation variance events during the progression from primary to metastatic colorectal tumors. Specifically, the metastatic sample presented an additional set of *de novo*-formed HMRs that covered 4% of the genome (Figure 4a). However, the biological relevance of these mainly partially methylated domains is poorly understood.

To pinpoint variation in DNA methylation that is relevant to regulation, we analyzed the variation occurring at HMRs and assessed their potential role in tumorigenesis. Initially, we determined the epigenetic variation present in c-HMRs, assuming that these loci regulate housekeeping and TSGs. Intriguingly, we noted a substantial increase in variation and DNA methylation level in c-HMRs over all cancer types (47.9%, s.d.>0.1) compared with the normal samples (11.2%, s.d.>0.1; Figures 4b and c). Within hypermethylated c-HMRs, we detected previously reported frequent DNA hypermethylation at gene promoters such as *MGMT* in glioblastoma, *NSD1* in neuroblastoma, *ESR1* in breast cancer, *GSTP1* in prostate cancer and the promoter hypermethylation of *RASSF1* in various cancer contexts (Figure 4d, Supplementary Figures S16a–d). Despite noting a global loss of DNA methylation, regions flanking c-HMR did not present noticeable differences in DNA methylation levels, indicating that these loci are protected from global hypomethylation (Figure 4c). We had previously described a similar phenomenon in a *DNMT3B* mutant ICF patient.^37^ Despite losing 41% of DNA methylation genome-wide, regions flanking CpG-rich promoters maintained their architecture, suggesting that the HMR structure is important for its regulatory activity.

An important aspect of our approach is that it was able to identify additional aberrations beyond the promoter context that may be involved in altered transcriptional regulation. By screening potential regulatory regions of established cancer genes (COSMIC Cancer Gene census), we determined, for example, that a c-HMR upstream of the TSG and DNA repair pathway component *FANCC* was hypermethylated in various cancer types (Figure 4e), results that were validated using the DNA methylated array-based approach and hundreds of cancer samples (Supplementary Figure S10). Subsequently, we aimed to assess the extent to which the epigenetic cell identity is preserved in cancer samples. Unlike the structural addition observed for c-HMRs, t-HMRs of related cancer types frequently lost their unique characteristics (Supplementary Figure S17). Substantial variation was observed between t-HMRs in cancer samples from unrelated tissue types, resulting in an inappropriate loss of DNA methylation in a non-tissue-specific manner (Figure 4b). The loss of epigenetic control of tissue-restricted gene expression may contribute to the loss of cell identity and the greater variability of expression seen in tumor cells.^10^

## Discussion

DNA methylation is altered in cancer. However, owing to the lack of genome-wide profiling studies, we have so far only been able to speculate about its magnitude. Therefore, we comprehensively profiled the DNA methylation landscape at base-pair resolution of numerous normal samples and cancer samples representing the most frequent cancer types. In line with previous studies using equivalent technologies, we observed a general consistency of DNA methylation levels in a large proportion of normal somatic cells.^3^ The variation in DNA methylation was very much greater in tumor samples in which two-thirds of the genome was affected by potential consequences for gene expression^10^ and tumor cell heterogeneity.^38^ From a functional perspective, we identified distinct HMRs with common and tissue-specific characteristics, which are probably essential for cell maintenance and cell-type-specific functions, respectively. Both types were greatly altered in the cancer context, with probable consequences for cell integrity and identity.^39^ In particular, DNA hypermethylation proved to be a very frequent event in all cancer types, and is probably involved in neoplastic transformation as a disease-driving event. Genetically defined cancer genes were epigenetically silenced in an unselective or cancer-type-specific manner. Potentially positively selected alterations were consistently mutually exclusive, suggesting that they actively contribute to neoplastic transformation and progression.^38, 39^

Overall, our study identified a large-scale loss and a focal gain of DNA methylation as being a hallmark of human cancer based on the extensive characterization of numerous normal and cancer samples. The provided cancer DNA methylomes at the single base-pair resolution highlight the massive and, at the same time, specific aberrant DNA methylation changes that occur in tumorigenesis and suggest that they are likely to contribute to the onset, progression and dissemination of the disease.

## Materials and methods

### Cell lines and primary tumor samples

Cancer cell lines were provided by the American Type Culture Collection (ATCC, Manassas, VA, USA). All cell lines were characterized by short tandem repeat analysis profiling (LGS Standards SLU) within 6 months after receipt. Cell lines were also tested for mycoplasma contamination. The used primary samples received approval by the corresponding ethic committees. The Clinical Research Ethics Committee of the Bellvitge University Hospital approved the current study under the reference PR055/10. All patients who supplied the primary tumor samples have given written informed consent. The experimental methods comply with the Helsinki Declaration.

### DNA methylation analyses

WGBS was performed as previously described.^8^ Genomic DNA libraries were constructed using the TruSeq Sample Preparation kit (Illumina Inc., San Diego, CA, USA). Following adaptor ligation, DNA was treated with sodium bisulfite using the EpiTect Bisulfite kit (Qiagen, Hilden, Germany). Adaptor-ligated DNA was enriched using the PfuTurboCx Hotstart DNA polymerase (Stratagene, San Diego, CA, USA). Paired-end DNA sequencing (two 100 bp reads) was developed using Illumina Hi-Seq 2000. WGBS was performed *de novo* for 9 samples and for the remaining 13 we used the reads available from our previous determination.^8^ Sequencing quality was determined by the Illumina Sequencing Analysis Viewer and the FastQC software (Babraham Bioinformatics, Cambridge, UK). Positional quality along the reads was confirmed, and biases toward specific motifs or GC enriched regions were excluded. Sequences alignment and DNA methylation calling was developed using Bismark software V.0.7.4 (Babraham Bioinformatics, Cambridge, UK).^40^ SAM/BAM and BED files handling was perfomed by SAMtools,^41^ bedtools^42^ and Tabix.^43^ Statistical analysis and graphic representation was obtained with R (http://www.R-project.org), libraries multicore and ggplot2. We smoothed the DNA methylation as previously described.^44^ Principal component analysis was carried out with the R princomp routine. Circular plots were obtained using CIRCOS software (Vancouver, BC, Canada).^45^ HG19 was used as reference genome and retrieved genomic information from Biomart (Hinxton, Cambridgeshire, UK)^46^ and Gencode V.16.^47^ Differentially methylated regions were identified screening for regions presenting more than five consecutive CpG sites that showed a consistent difference between the 95% confidence interval of the smoothed methylation profile.^44^ HMRs were identified as previously described.^23^ The source code is available at GitHub (https://github.com/mesteller-bioinfolab/meth_map_cancer.git). For the identification of HMRs common to all analyzed tissue types (c-HMRs), we obtained the genomic regions that were strictly covered by HMRs in all normal tissues. Further, we merged regions with a maximal genomic distance of 100 bp. t-HMRs were defined as HMRs that did not present any overlap with HMR identified in other normal tissue types. We defined frequent HMRs as HMRs present in >50% of the normal tissues analyzed and not overlapping c-HMRs. Infinium HumanMethylation450 BeadChip assays were performed as previously described^8, 27^ and the data was processed using the Bioconductor minfi package (Baltimore, MD, USA).^48^ Hierarchical clustering was computed using Jaccard distance and the Ward clustering method. In the cases continuous data points (that is, average DNA methylation), the values were dichotomized. In order to validate the WGBS-associated t-HMRs, we computed the array-based average DNA methylation level of the t-HMRs tissue-wise. Then, given a threshold, true positives events were assigned when the average DNA methylation was below the threshold for the corresponding tissue. False positives were assigned when the average DNA methylation was below the threshold for samples of unrelated tissue origin. DNA methylation and expression data are available at the SRA and GEO repositories under accession numbers SRP033252, GSE76269, GSE56763, GSE39279 and GSE52272. The source code of all computational analysis is available at GitHub (https://github.com/mesteller-bioinfolab/meth_map_cancer.git).

### Mutual exclusivity analysis

A mutually exclusive alteration pattern reflects that the genes are selectively targeted by cancer, as more than a single hit does hypothetically not provide any further benefit for the tumor cell and the tumorigenic process. The significance of the mutually exclusive pattern has been measured by the empirical *P*-value obtained from comparing the coverage of the observed gene set (that is, the number of samples exhibiting methylation alterations across these genes) to a background model obtained by 1 × 10^5^ simulations. Each simulation was performed by a random generation of altered samples per gene in which the number of observed number of altered samples per gene and the overall observed alteration burden per sample was respected by assigning weights to the alteration probability in each of them, following the rationale of the CDOCOCA method.^49^ The method is available in the Gitools software (Barcelona, Catalonia, Spain).^50^

## Figures and Tables

**Table 1:** Whole-genome bisulfite sequencing of 22 human samples

*Sample ID*	*Status*	*Tissue*	*Origin*	*Total reads*	*Coverage (fold)*	*AVR Meth.*	*# HMRs*	*Coverage (bp)*	*Coverage (%)*	*Average size (bp)*
CD19	Normal	B cells	Primary	318 714 023	6	76	56 894	56 552 703	1.9	655
Brain_G	Normal	Brain (gray matter)	Primary	275 099 021	5.7	74.9	46 693	54 771 999	1.8	783
Brain_W	Normal	Brain (white matter)	Primary	557 237 398	11.1	77.1	58 215	67 842 262	2.2	809
Breast	Normal	Breast	Primary	606 872 747	15.1	73	63 648	77 291 411	2.5	871
Colon	Normal	Colon	Primary	609 043 678	13.7	69.6	45 020	58 103 004	1.9	925
Liver	Normal	Liver	Primary	1 003 013 615	25.2	73.2	54 545	75 779 635	2.5	957
Lung	Normal	Lung	Primary	333 333 332	7.2	74.4	43 259	48 772 726	1.6	748
Placenta	Normal	Placenta	Primary	447 848 405	10.4	58.2	76 898	586 617 157	19.3	4942
Prostate	Normal	Prostate	Primary	702 575 404	17.3	74.5	66 711	81 099 795	2.7	899
Colon_P	Cancer	Colorectal cancer	Primary	670 281 443	16.7	66.5	71 774	181 767 349	6.0	1902
Colon_M	Cancer	Colorectal cancer metastasis	Primary	652 566 967	16.3	62.4	86 810	330 654 110	10.9	2825
Liver_P1	Cancer	Liver carcinoma	Primary	658 876 767	16.2	50.6	72 633	558 373 155	18.4	4919
Liver_P2	Cancer	Liver carcinoma	Primary	718 665 563	17.5	64.6	43 965	124 483 271	4.1	1274
468PT	Cancer	Breast cancer	Cell line	626 288 553	15.4	57.1	186 645	873 241 247	28.8	2710
468LN	Cancer	Breast cancer metastasis	Cell line	600 134 926	14.3	42.8	192 305	1 081 026 523	35.6	3646
U87MG	Cancer	Glioma	Cell line	281 524 883	6.3	55.7	78 444	660 392 120	21.7	4682
SH5YSY	Cancer	Neuroblastoma	Cell line	277 166 468	6.2	65.4	85 756	586 978 640	19.3	3614
PC3	Cancer	Prostate cancer	Cell line	697 835 354	17.8	54.2	141 730	779 429 448	25.7	2826
22RV1	Cancer	Prostate cancer	Cell line	465 560 242	11.4	55.2	102 806	701 717 497	23.1	3715
H1437	Cancer	Lung adenocarcinoma	Cell line	333 333 332	7.9	48.1	106 188	1 059 922 017	34.9	6484
H1672	Cancer	Small cell lung cancer	Cell line	329 691 560	7.4	65.6	87 979	513 193 022	16.9	3016
H157	Cancer	Squamous cell cancer	Cell line	333 333 332	7.8	41.8	107 206	1 159 333 666	38.2	6942

Abbreviation: HMR, hypomethylated region.
