# Supplementary material for: A DNA methylation map of human cancer at single base-pair resolution
Source: Oncogene. 2017 Jun 5;36(40):5648–57. doi: 10.1038/onc.2017.176 (PMC5633654; doi:10.1038/onc.2017.176)
Supplement: Supplementary Information [file onc2017176x1.pdf]

Supplemental Information

Supplemental figure legends: Figures S1-S17

Supplemental tables: Tables S1-S3

Supplemental figure legends

Figure S1. Principle component analysis (PCA) separated normal tissues, primary tumor samples and cancer cell lines. PCA of CpG methylation levels obtained from WGBS of 22 human samples. Tissues of origin are color-coded and matched samples are indicated (bold line). Distinct sample sources are highlighted (circled).

Figure S2. Repetitive elements loose DNA methylation in cancer samples. Average DNA methylation levels of repetitive elements (RepeatMasker) obtained from WGBS of normal tissues and cancer samples.

Figure S3. Gradient genome-wide loss of DNA methylation level and integrity from normal tissue to cancer metastasis. (a) Whole-genome representation of DNA methylation levels (5Mbp windows) of normal breast tissue and donor-matched primary and metastatic breast cancer samples. (b) Correlation of DNA methylation levels between neighboring CpG sites in normal breast, primary tumor and metastasis.

Figure S4. Active regulatory region frequently overlap with hypomethylated regions (HMRs). Frequency of histone mark defined regulatory regions overlapping with HMRs in normal breast, B-cells and liver tissue.

Figure S5. Hypomethylated regions (HMRs) in house-keeping gene promoters. (a-f) Representative HMRs overlapping promoters of the housekeeping genes. Smoothed (upper panel) and base-pair resolution (lower panels) representation display DNA methylation levels of 9 normal tissue samples. Transcription start site (broken line) and direction (+/-) are indicated.

Figure S6. Hypomethylated regions (HMRs) distal to promoters enriched in tissue specific target genes. Enrichment analysis in biological processes for t-HMRs distal to gene promoters (<10kb, Bonferroni adjusted p-value).

Figure S7. Validation of WGBS-derived HMR in 114 normal tissue samples. (a) Percentage of validated c-HMRs as a function of the threshold applied on the average HMR methylation measured by the HumanMethylation450 BeadChip (solid lines). Corresponding thresholds to validate 95% of c-HMRs is indicated per tissue type.

Dashed horizontal line spots 95% of c-HMRs validated. (b) ROC curves for the validation of t-HMR (solid lines). The Area Under the Curve (AUC) per tissue type is indicated.

Figure S8. Hierarchical clustering using t-HMRs splits normal samples into tissue of origin. Hierarchical cluster of 114 normal samples and DNA methylation levels from 9,391 t-HMRs interrogated by the BeadChip. Average HMR methylation levels are gradiently color-coded from 0% (light blue) to 100% (dark blue).

Figure S9. Hypermethylation of common HMRs (c-HMRs) 1,112 primary cancer samples. (a) Frequency of hypermethylation of c-HMRs in 1,112 cancer samples analyzed using the HumanMethylation450 BeadChip. Dashed line separates c-HMRs with high (>25% of samples) hypermethylation frequencies. (b) Variance profile of c-HMRs, inside and outside promoter regions, of 1,112 samples split by cancer type.

Figure S10. Genome-scale DNA methylation analysis of 1,112 cancer samples revealed cancer type specific signatures outside the promoter context. (a) Unsupervised hierarchical clustering of 1,112 cancer samples and 3,236 hypermethylated HMRs outside gene promoters using Jaccard distances and the Ward cluster method. Average HMR methylation levels are gradiently color-coded from 0% (light blue) to 100% (dark blue). (b) Frequently hypermethylated HMRs outside promoters near tumor-suppressor genes (<10kb) of 1,112 samples ordered by cancer type. HMR methylation status is categorized in hyper- (>33% DNA methylation, dark blue) and hypomethylated (<33% DNA methylation, light blue).

Figure S11. Proportion of c-HMRs frequently gaining DNA methylation in cancer stratified by regulatory segments defined in the respective normal tissues. The analysis is split by normal-cancer type pair and each point and error bar (95 % CI) represent one segmentation category.

Figure S12. DNA hypermethylation revealed mutual exclusivity in cancer pathways. (a) DNA hypermethylation events in promoter associated c-HMRs (black boxes) of genes related to Non-small cell lung cancer pathogenesis (KEGG). Selected genes show significant patterns of mutual exclusivity (empirical p<0.01). Displayed are hypermethylation events in lung adenocarcinoma (LAD, purple) and lung squamous cell carcinomas (LSQ, blue). (b) Mutual exclusivity analysis of recurrent hypermethylation events in PIK3 subunits (*PIK3R1*, *PIK3R2*, *PIK3R5*) in LAD (purple),

small-cell lung cancer (SCLC, pink) and LSQ (blue). (c) Genes involved in chemical induced carcinogenesis (KEGG) revealed significant (empirical $p<0.01$) mutually exclusive patterns in LAD (purple), LSQ (blue) and SCLC (pink).

Figure S13. Cancer type specific hypermethylation of gene promoters. DNA hypermethylated putative tumor-suppressor genes with tissues specific patterns (OR>30). Tumor-suppressor genes were defined by recurrent alterations including mutation (blue), hypermethylation (red) or deletion (orange) events in cancer.

Figure S14. DNA methylation alterations in human cancer samples. Size distribution and DNA methylation levels of total HMRs in normal tissues (a), primary tumors (b), metastatic progression (c) and cancer cell lines (d). Densities are displayed from low (blue) to high (red).

Figure S15. Distribution of HMRs analyzed by MethylSeekR. Average DNA methylation of HMRs (y-axis) and size (x-axis) in normal colon (left panel), primary tumor (central panel) and metastasis (right panel) as classified by MethylSeekR. Regions are split in Partially Methylated Domains (PMD), Low Methylation Regions (LMR), Unmethylted regions (UMR) or only classified previously as HMR. Each pixel represents an HMR region. PMD detection was not performed on the normal colon sample.

Figure S16. Promoter hypermethylation of cancer genes identified by WGBS. Promoter methylation profiles derived from WGBS data identified hypermethylation of (a) *MGMT* in glioblastoma (U87MG), (b) *NSD1* in neuro- and glioblastoma (SH5YSY, U87MG), (c) *GSTP1* in prostate cancer (22RV1) and (d) *MLH1* in matched primary and metastatic colorectal cancer samples.

Figure S17. Profound changes in the DNA methylation structure at tissue-specific HMRs (t-HMRs) in cancer samples. DNA methylation structure of t-HMRs in normal (a) and cancer (b) samples. CpG methylation levels are averaged in 4bp windows over all c-HMRs and displayed +/- 2kb flanking the HMR center. For cancer samples only tissue-matched t-HMRs are displayed.

Figure S1

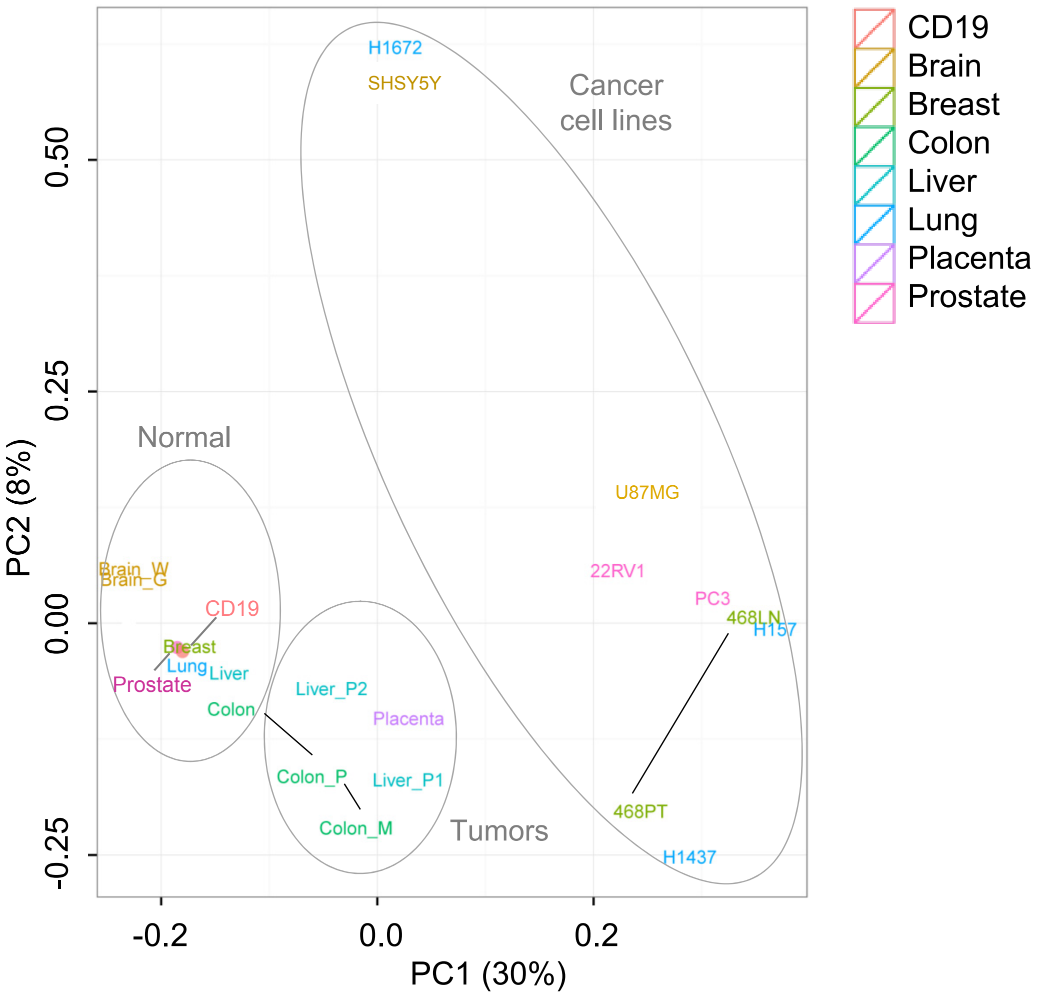

CD19
Brain
Breast
Colon
Liver
Lung
Placenta
Prostate
Cancer cell lines
Normal
Tumors
H1672
SHSY5Y
U87MG
22RV1
PC3
468LN
H157
468PT
H1437
Brain_W
Brain_G
CD19
Breast
Lung
Liver
Prostate
Colon
Liver_P2
Placenta
Colon_P
Liver_P1
Colon_M
0.50
0.25
0.00
-0.25
-0.2
0.0
0.2
PC2 (8%)
PC1 (30%)

Figure S2

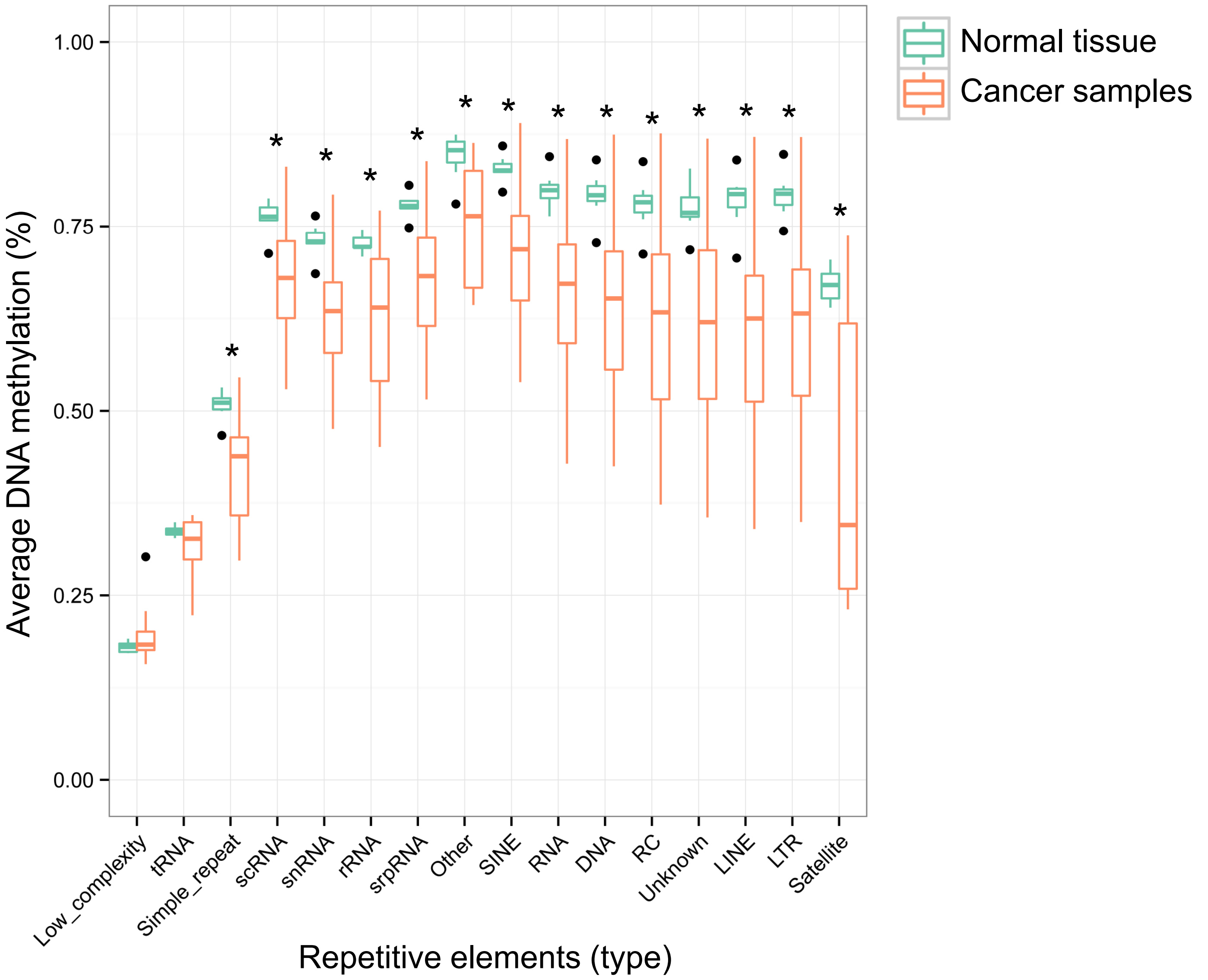
Normal tissue
Cancer samples
Average DNA methylation (%)
1.00
0.75
0.50
0.25
0.00
Low_complexity
tRNA
Simple_repeat
scRNA
snRNA
rRNA
srpRNA
Other
SINE
RNA
DNA
RC
Unknown
LINE
LTR
Satellite
Repetitive elements (type)

Figure S3

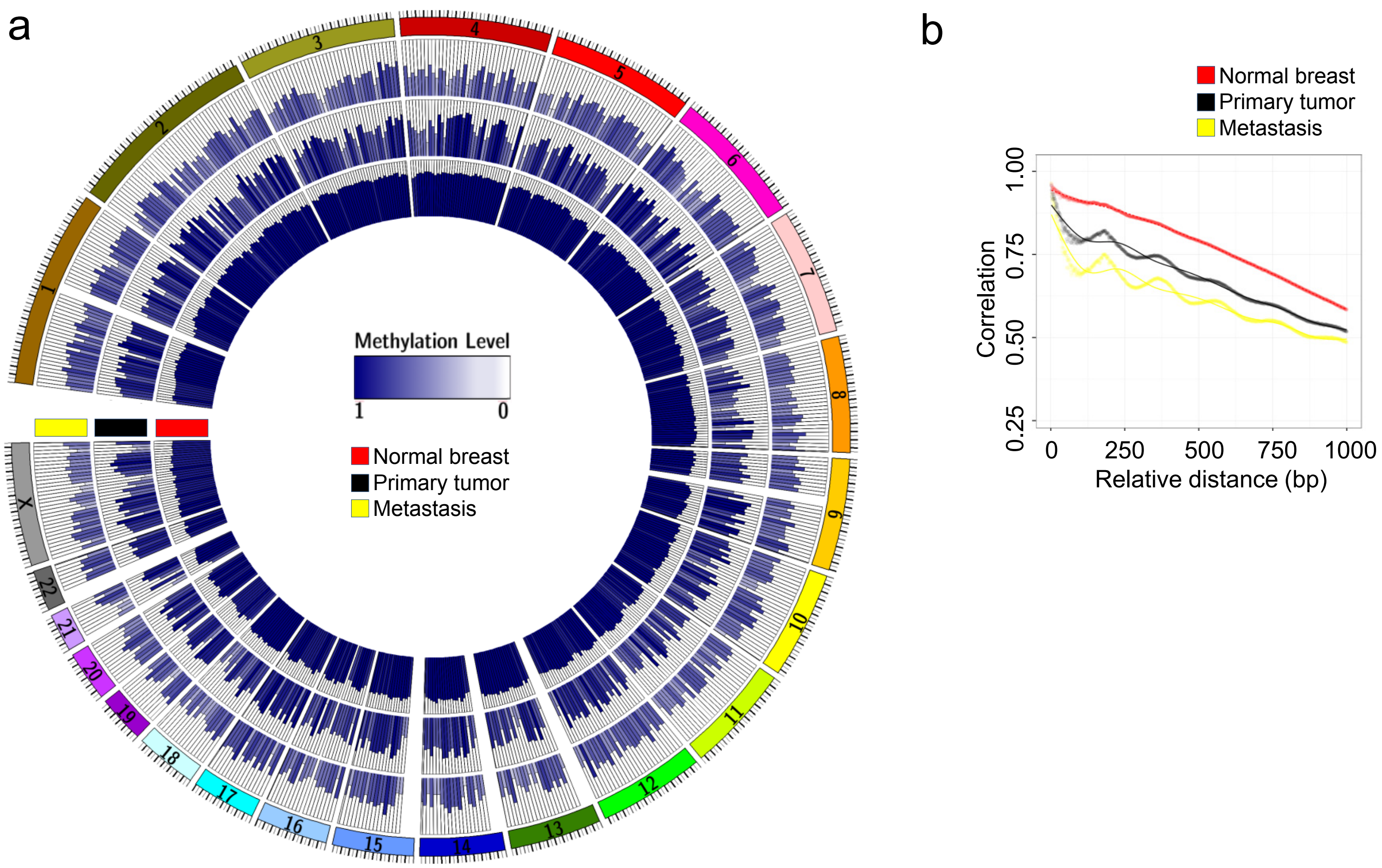
a
Methylation Level
1
0
Normal breast
Primary tumor
Metastasis
1
2
3
4
5
6
7
8
9
10
11
12
13
14
15
16
17
18
19
20
21
22
X
b
Normal breast
Primary tumor
Metastasis
Correlation
0.25
0.50
0.75
1.00
0
250
500
750
1000
Relative distance (bp)

Figure S4

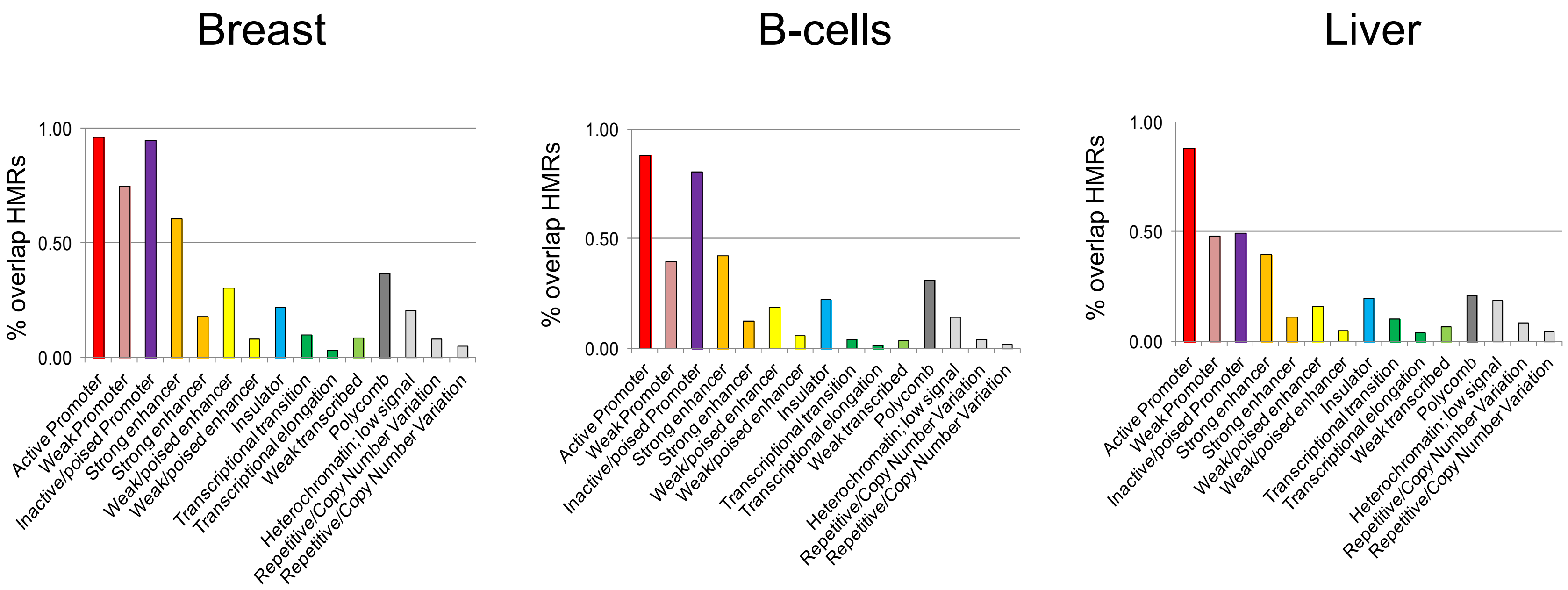
Breast
B-cells
Liver
% overlap HMRs
1.00
0.50
0.00
Active Promoter
Weak Promoter
Inactive/poised Promoter
Strong enhancer
Strong enhancer
Weak/poised enhancer
Weak/poised enhancer
Insulator
Transcriptional transition
Transcriptional elongation
Weak transcribed
Polycomb
Heterochromatin; low signal
Repetitive/Copy Number Variation
Repetitive/Copy Number Variation

Figure S5

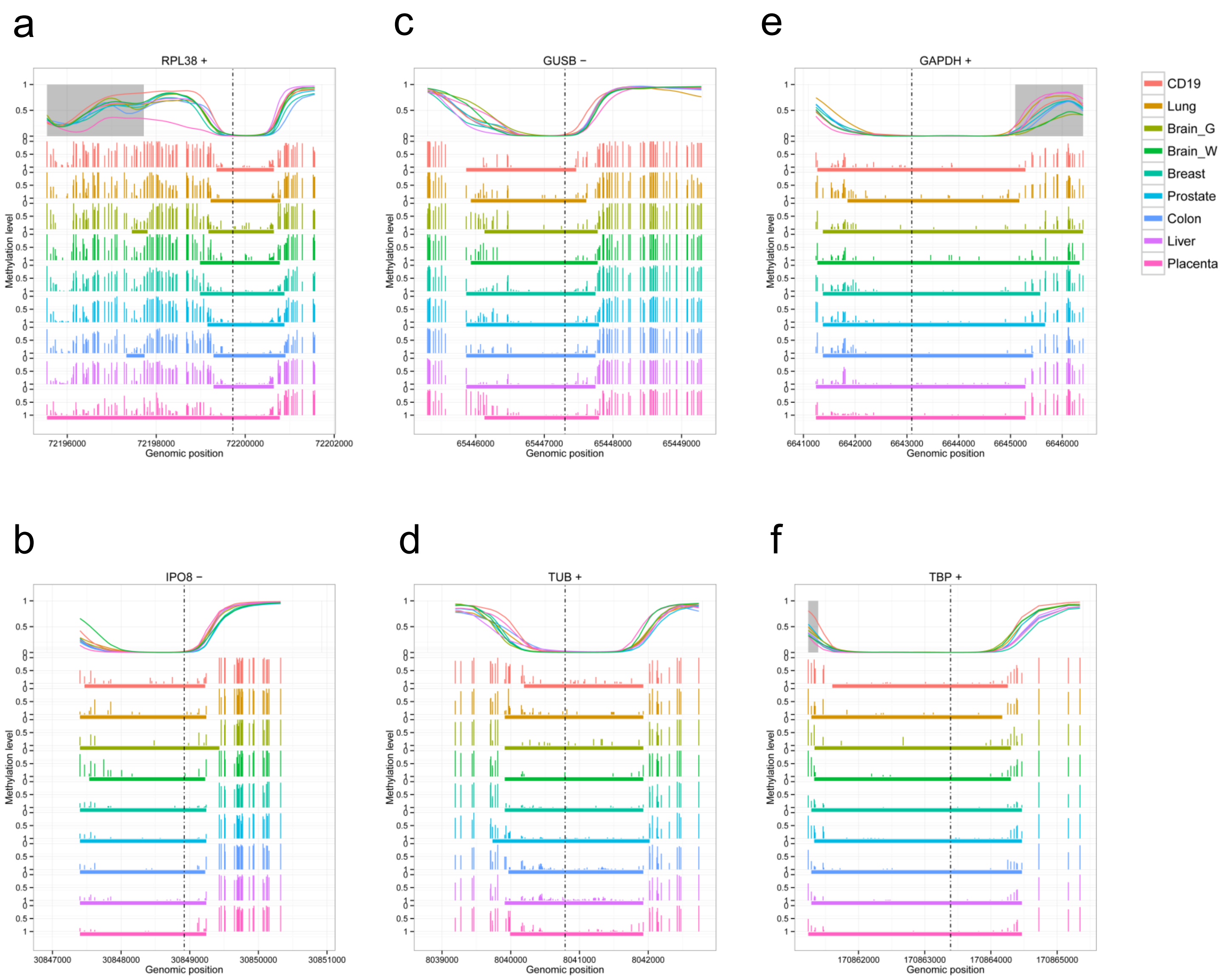

a
RPL38 +
Methylation level
Genomic position
72196000
72198000
72200000
72202000
c
GUSB −
65446000
65447000
65448000
65449000
e
GAPDH +
6641000
6642000
6643000
6644000
6645000
6646000
b
IPO8 −
30847000
30848000
30849000
30850000
30851000
d
TUB +
8039000
8040000
8041000
8042000
f
TBP +
170862000
170863000
170864000
170865000
CD19
Lung
Brain_G
Brain_W
Breast
Prostate
Colon
Liver
Placenta

Figure S6

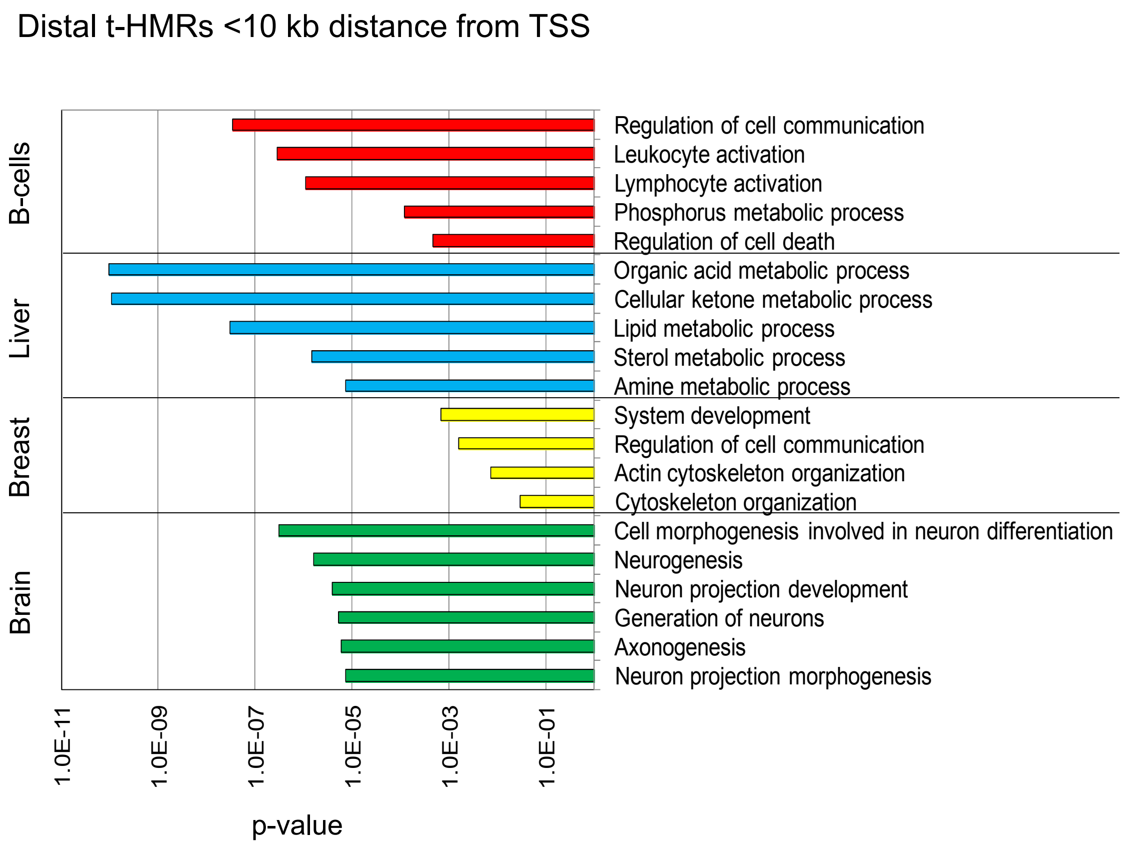

Distal t-HMRs <10 kb distance from TSS
B-cells
Liver
Breast
Brain
Regulation of cell communication
Leukocyte activation
Lymphocyte activation
Phosphorus metabolic process
Regulation of cell death
Organic acid metabolic process
Cellular ketone metabolic process
Lipid metabolic process
Sterol metabolic process
Amine metabolic process
System development
Regulation of cell communication
Actin cytoskeleton organization
Cytoskeleton organization
Cell morphogenesis involved in neuron differentiation
Neurogenesis
Neuron projection development
Generation of neurons
Axonogenesis
Neuron projection morphogenesis
1.0E-11
1.0E-09
1.0E-07
1.0E-05
1.0E-03
1.0E-01
p-value

Figure S7

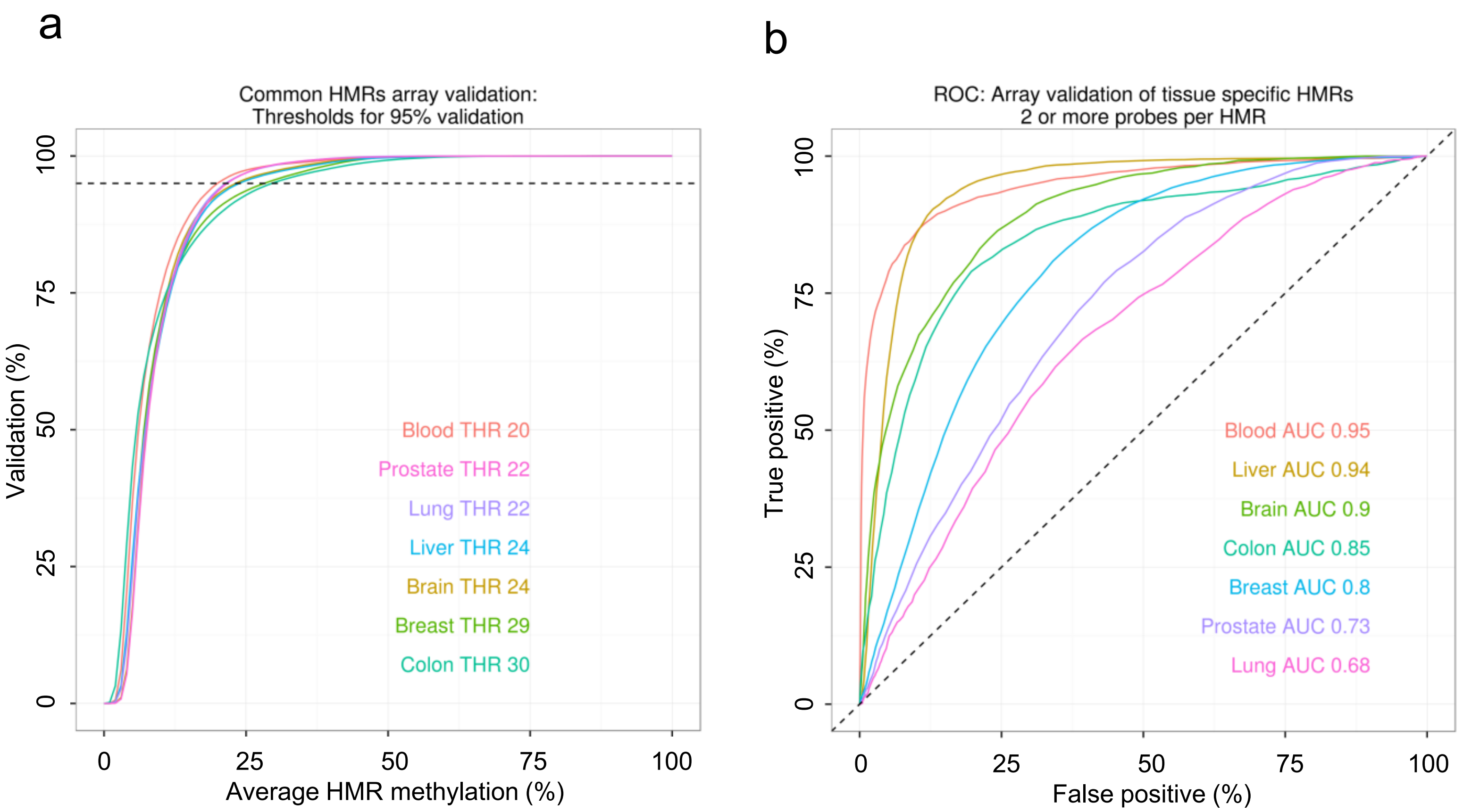
a
Common HMRs array validation:
Thresholds for 95% validation
Validation (%)
Average HMR methylation (%)
0
25
50
75
100
Blood THR 20
Prostate THR 22
Lung THR 22
Liver THR 24
Brain THR 24
Breast THR 29
Colon THR 30
b
ROC: Array validation of tissue specific HMRs
2 or more probes per HMR
True positive (%)
False positive (%)
Blood AUC 0.95
Liver AUC 0.94
Brain AUC 0.9
Colon AUC 0.85
Breast AUC 0.8
Prostate AUC 0.73
Lung AUC 0.68

Figure S8

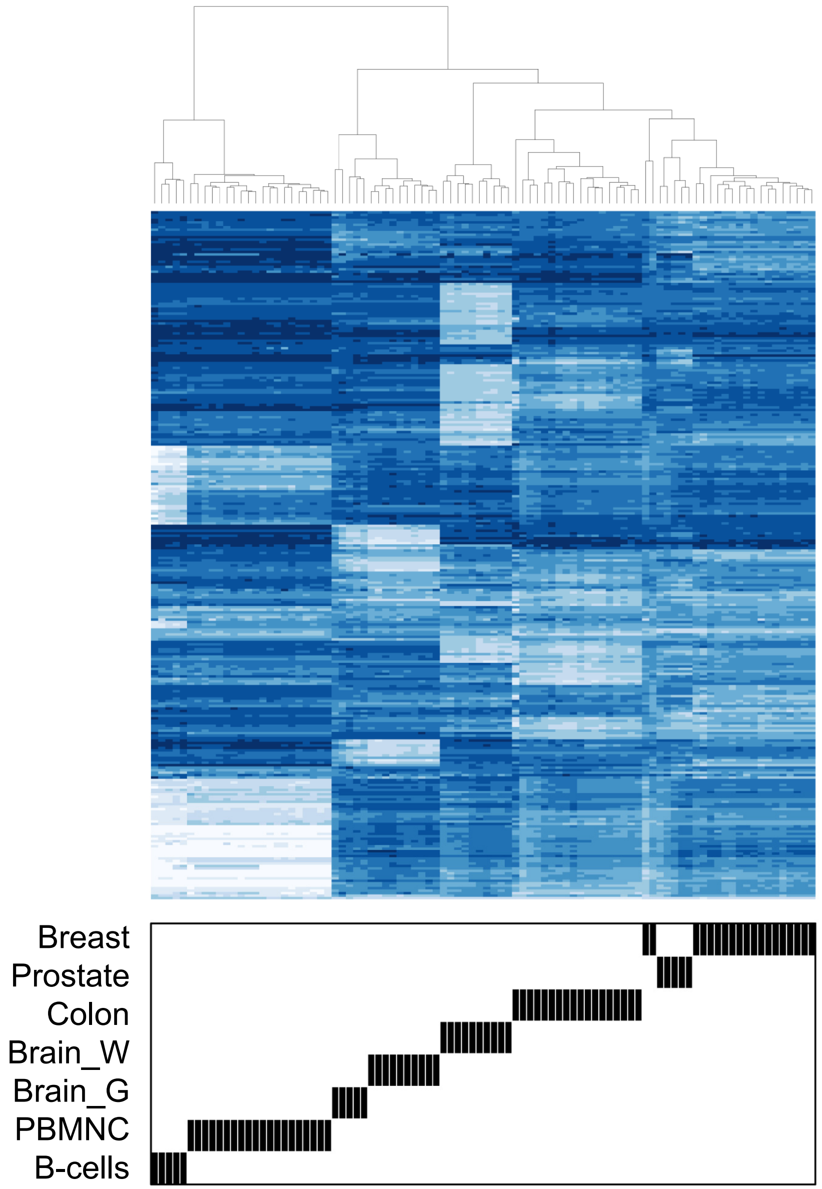

Figure S9

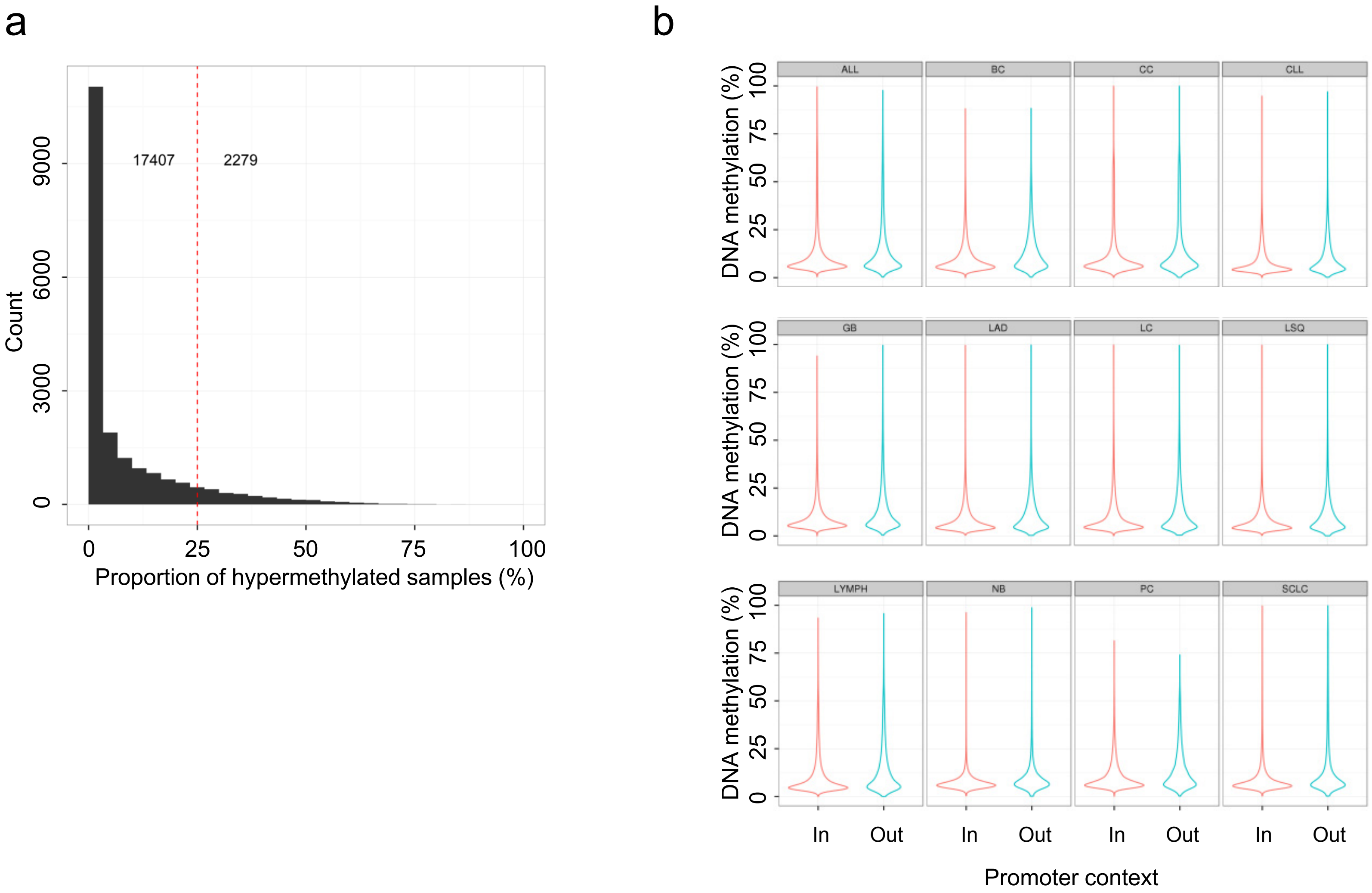
a
17407
2279
Count
0
3000
6000
9000
0
25
50
75
100
Proportion of hypermethylated samples (%)
b
ALL
BC
CC
CLL
GB
LAD
LC
LSQ
LYMPH
NB
PC
SCLC
DNA methylation (%)
0
25
50
75
100
In
Out
Promoter context

Figure S10

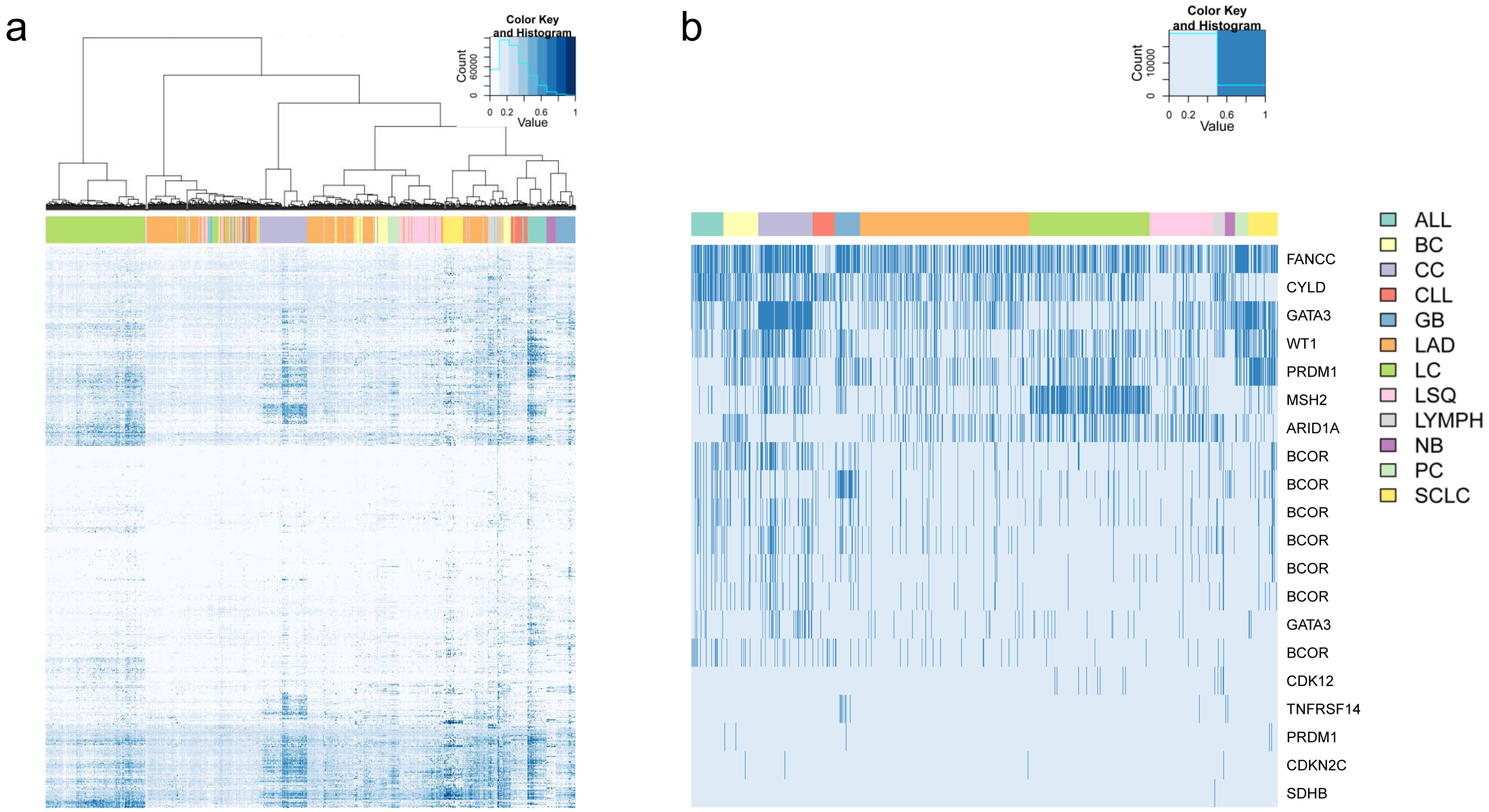
a
Color Key and Histogram
Count
0
60000
0
0.2
0.6
1
Value
b
Color Key and Histogram
Count
0
10000
0
0.2
0.6
1
Value
FANCC
CYLD
GATA3
WT1
PRDM1
MSH2
ARID1A
BCOR
BCOR
BCOR
BCOR
BCOR
BCOR
GATA3
BCOR
CDK12
TNFRSF14
PRDM1
CDKN2C
SDHB
ALL
BC
CC
CLL
GB
LAD
LC
LSQ
LYMPH
NB
PC
SCLC

Figure S11

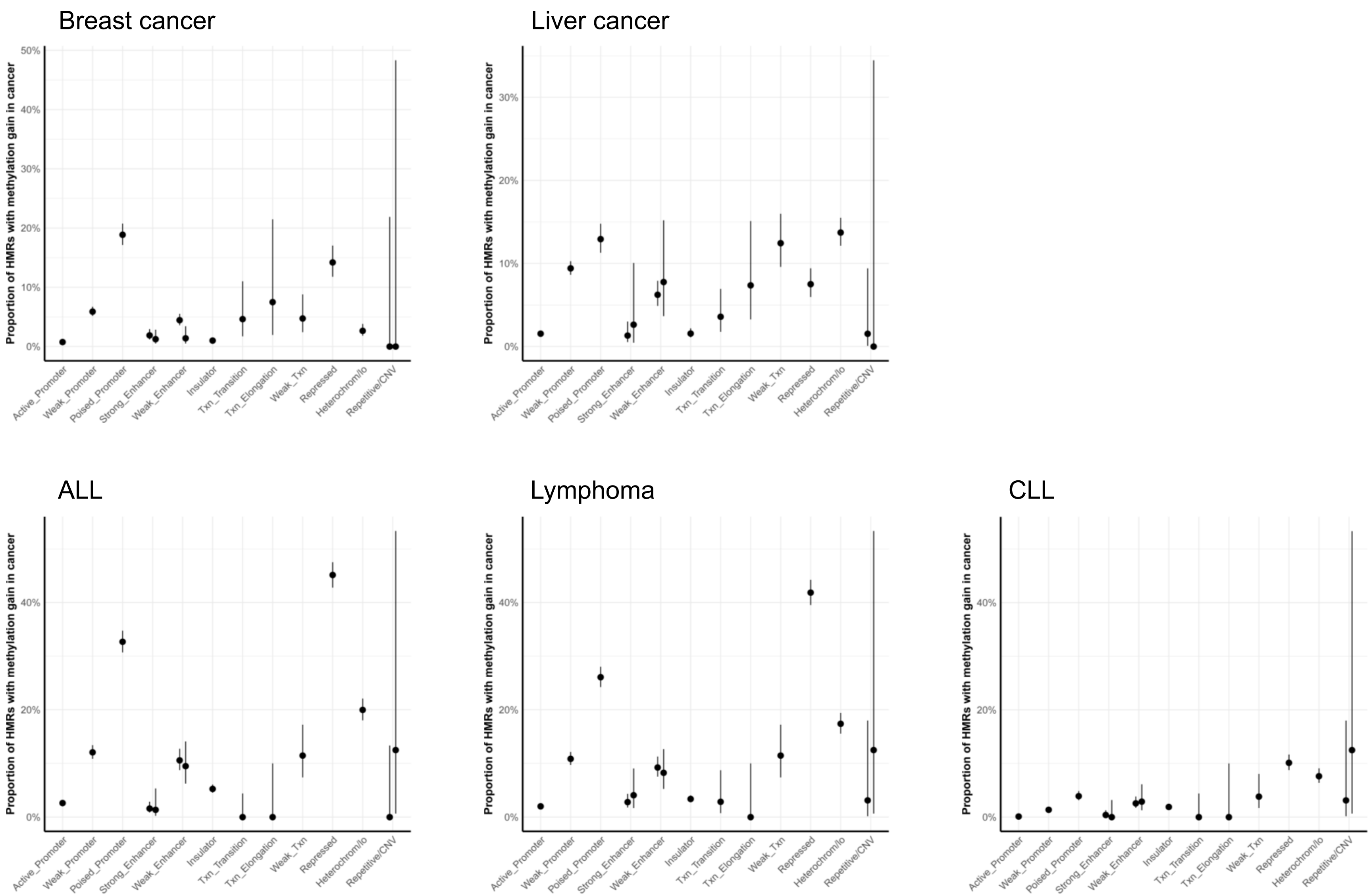

Breast cancer
Liver cancer
ALL
Lymphoma
CLL
Proportion of HMRs with methylation gain in cancer
0%
10%
20%
30%
40%
50%
Active_Promoter
Weak_Promoter
Poised_Promoter
Strong_Enhancer
Weak_Enhancer
Insulator
Txn_Transition
Txn_Elongation
Weak_Txn
Repressed
Heterochrom/lo
Repetitive/CNV

Figure 12

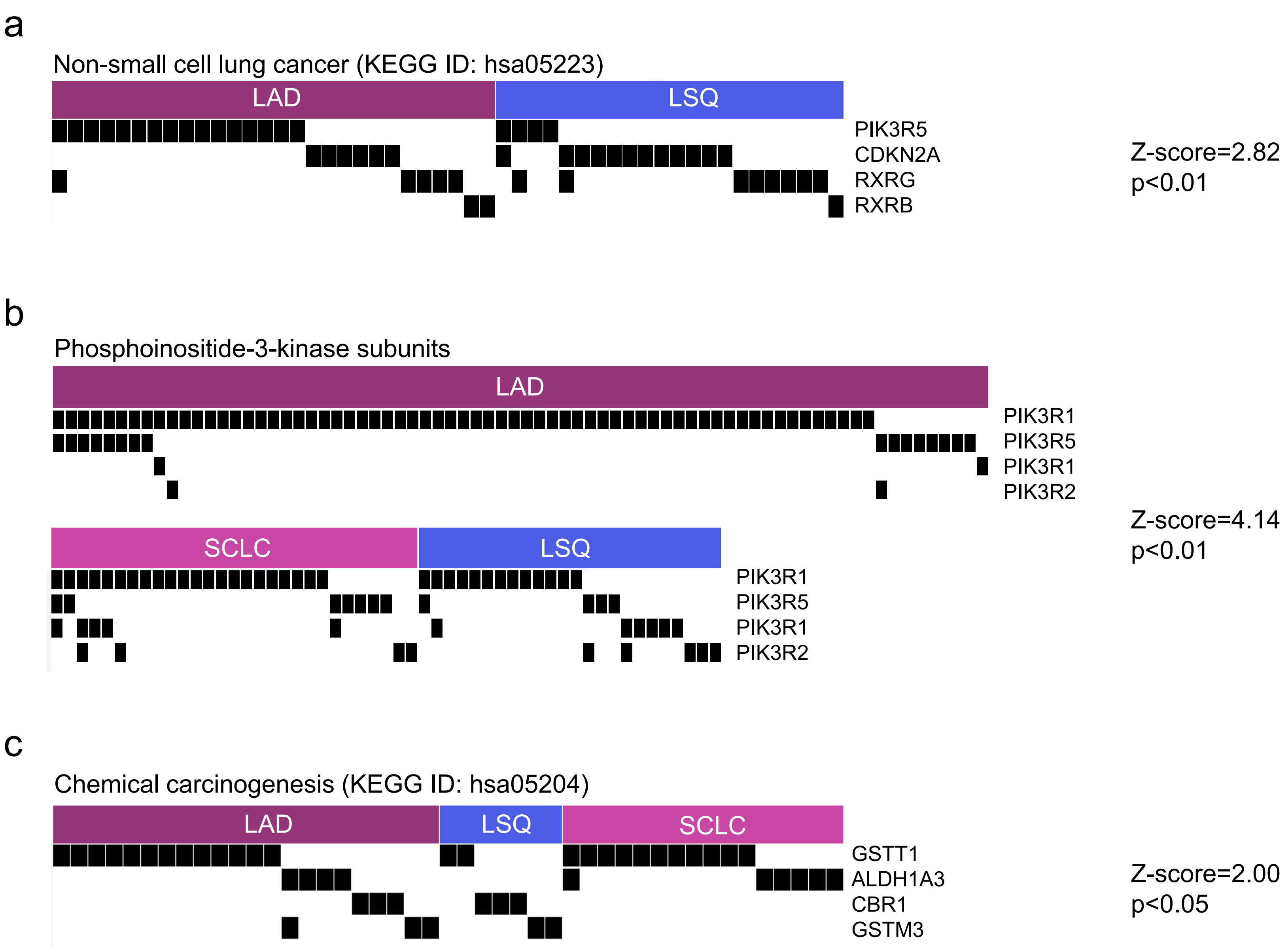

a
Non-small cell lung cancer (KEGG ID: hsa05223)
LAD
LSQ
PIK3R5
CDKN2A
RXRG
RXRB
Z-score=2.82
p<0.01
b
Phosphoinositide-3-kinase subunits
LAD
PIK3R1
PIK3R5
PIK3R1
PIK3R2
SCLC
LSQ
PIK3R1
PIK3R5
PIK3R1
PIK3R2
Z-score=4.14
p<0.01
c
Chemical carcinogenesis (KEGG ID: hsa05204)
LAD
LSQ
SCLC
GSTT1
ALDH1A3
CBR1
GSTM3
Z-score=2.00
p<0.05

Figure 13

CC	LYMPH	LC	GBM	BC	LAD	PC	SCLC	NB	LSQ	Cancer gene
333.65	1.55	0.0	0.0	0.0	0.0	0.0	0.0	0.0	0.0	CADM2
317.56	0.0	0.12	0.0	0.0	0.0	0.0	0.0	0.0	0.0	ERBB4
317.56	0.0	0.12	0.0	0.0	0.0	0.0	0.0	0.0	0.0	COL18A1
259.09	0.0	0.13	0.39	0.0	0.0	0.0	0.0	0.0	0.0	EPHA7
257.16	1.9	0.0	0.0	0.0	0.0	0.0	0.0	0.0	0.0	MMP2
253.86	1.39	0.32	0.19	0.13	0.019	0.0	0.0	0.0	0.0	AMPH
219.87	1.23	0.0	0.0	0.0	0.0	1.13	0.0	0.0	0.0	SYNE1
164.7	0.0	0.0	0.0	0.0	0.0	0.0	1.06	0.0	0.0	MN1
0.0	142.24	0.0	0.0	0.0	0.0	0.0	5.74	0.0	0.0	FAT1
124.18	0.82	0.20	0.37	0.0	0.038	0.0	0.0	0.0	0.0	ADCY1
119.66	1.1	0.17	0.0	0.0	0.0	0.0	0.43	0.0	0.0	LIFR
96.45	0.0	0.0	2.02	0.0	0.0	0.0	0.0	0.0	0.0	MGMT
96.45	0.0	0.0	0.0	0.0	0.0	0.0	1.71	0.0	0.0	EPM2AIP1, MLH1
0.20	0.0	0.0	74.32	0.0	0.099	0.0	1.8	19.65	0.17	EEF1A1
81.53	3.36	0.81	0.69	0.27	0.12	0.0	0.21	0.0	0.044	FBN2, SLC27A6
85.92	0.0	0.38	0.0	0.0	0.0	0.0	0.0	0.0	0.0	LCP1, LRRC63
85.92	0.0	0.0	0.0	0.0	0.24	0.0	0.0	0.0	0.0	FN1
75.63	5.54	0.0	0.0	0.0	0.0	0.0	0.0	0.0	0.0	LRRK2
71.29	1.0	0.0	0.95	0.32	0.0	0.0	0.80	0.0	0.17	CNTN4
70	0.0	0.38	1.09	0.0	0.0	0.0	0.0	0.0	0.0	PTPRD
0.0	0.0	0.0	62.91	0.0	0.0	0.0	5.74	0.0	0.0	CUL1
0.0	0.0	0.0	62.91	0.0	0.0	0.0	5.74	0.0	0.0	CASP8
0.33	54.31	0.0	0.0	0.0	0.61	7.92	1.37	0.0	0.92	CAST
0.0	0.0	1.14	62.91	0.0	0.0	0.0	0.0	0.0	0.0	PLEC
0.0	0.0	0.0	1.84	0.0	0.19	0.0	9.04	49.01	0.67	RPL22, RNF207
0.0	45.83	0.0	6.77	0.0	0.0	0.0	5.74	0.0	0.0	CDH1
0.0	45.83	0.0	6.77	4.81	0.0	0.0	0.0	0.0	0.0	FOXA2
53.55	0.0	0.0	0.0	0.0	0.18	0.0	1.43	0.0	0.0	MATK
1.93	0.0	0.13	2.87	1.26	0.21	2.95	4.95	39.08	1.0	CARD11
0.0	12.54	0.27	3.07	0.0	0.0	0.0	33.21	4.81	0.0	ERBB2
52.98	0.0	0.32	0.0	0.0	0.15	0.0	0.0	0.0	0.33	LMO1
2.95	45.83	0.0	0.0	4.81	0.0	0.0	0.0	0.0	0.0	LYN
0.45	29.85	0.0	1.66	0.36	0.11	13.89	6.66	0.0	0.59	NCOA4
0.0	0.0	0.0	2.89	0.0	0.0	0.0	30.69	19.05	0.0	KALRN
0.0	0.0	0.0	42.97	0.0	0.0	0.0	8.74	0.0	0.0	RILP, PRPF8
0.0	0.0	0.0	4.59	0.0	0.0	0.0	33.49	12.7	0.0	TGIF1
8.72	4.19	0.0	0.0	0.0	0.0	34.88	1.53	0.0	0.41	RARB
0.28	41.4	0.11	4.01	0.45	0.83	0.0	0.54	0.0	0.76	SEL1L2, MACROD2
0.0	43.81	3.43	0.0	0.0	0.0	0.0	0.0	0.0	0.0	KIAA1549
0.0	43.81	3.43	0.0	0.0	0.0	0.0	0.0	0.0	0.0	SPATA6
2.33	0.17	4.65	0.24	0.71	0.15	1.2	36.71	0.21	0.56	C9orf53, CDKN2A
0.0	10.28	0.0	1.39	3.19	0.044	0.0	29.98	0.0	0.68	FOXA2
40.79	1.4	0.22	1.35	0.0	0.0	0.0	1.14	0.0	0.0	AR
0.0	30.65	0.86	0.0	0.0	0.0	0.0	11.65	0.0	0.0	CHD3
4.28	3.88	0.0	0.0	0.31	0.28	0.0	30.88	0.0	0.72	PRDM13
35.53	0.58	0.42	0.0	0.71	0.15	0.0	0.58	0.0	1.63	ZNF132
0.0	0.0	0.93	0.0	1.1	0.0	0.0	35.86	0.0	0.57	MECOM
0.0	27.93	1.01	6.43	0.0	0.0	0.0	0.81	0.0	1.67	DNMT3A
31.23	0.43	0.0	0.19	0.44	0.28	0.0	4.35	0.0	0.30	WT1

Alteration

Figure S14

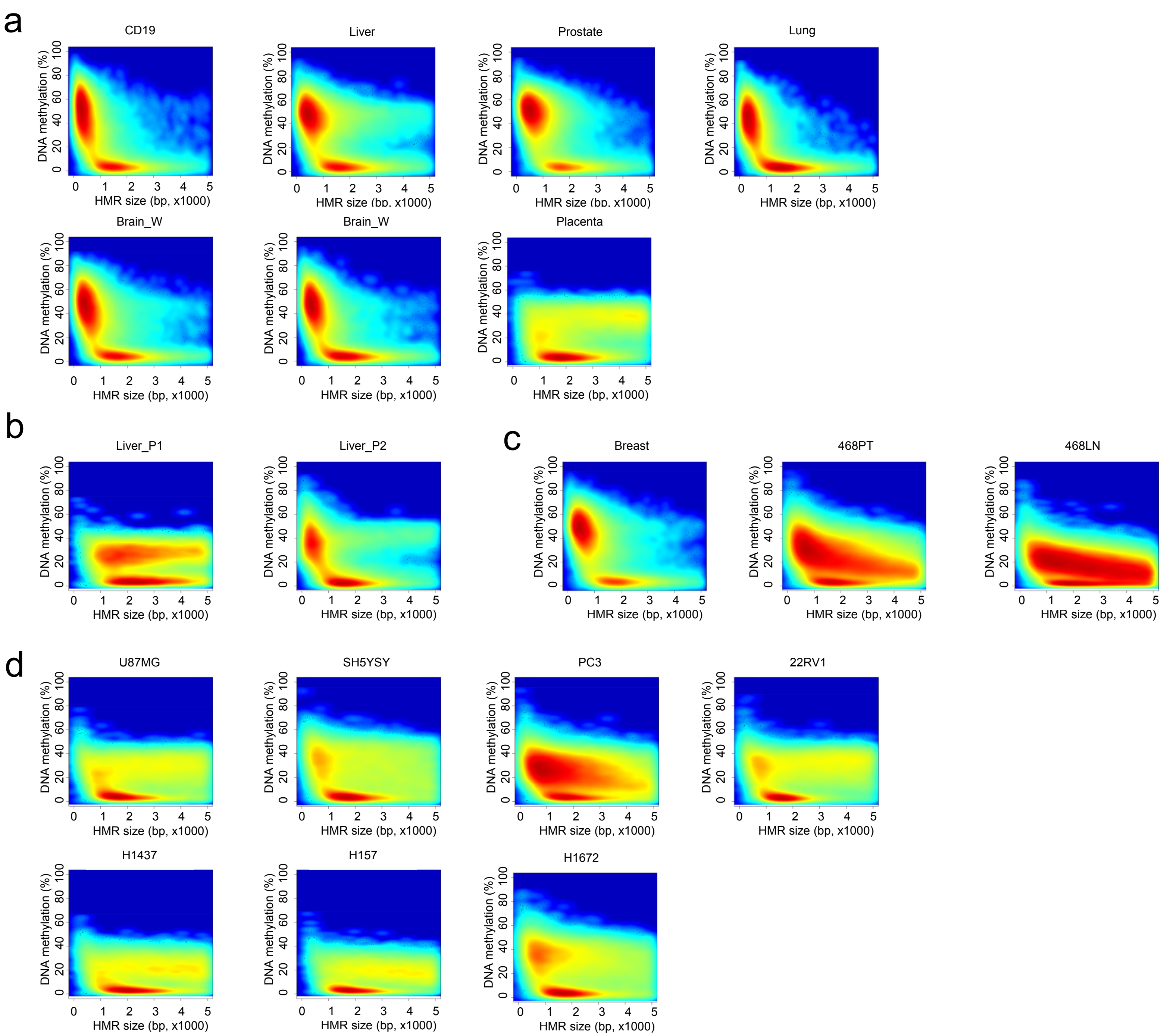
a
CD19
Liver
Prostate
Lung
Brain_W
Brain_W
Placenta
b
Liver_P1
Liver_P2
c
Breast
468PT
468LN
d
U87MG
SH5YSY
PC3
22RV1
H1437
H157
H1672
DNA methylation (%)
HMR size (bp, x1000)

Figure S15

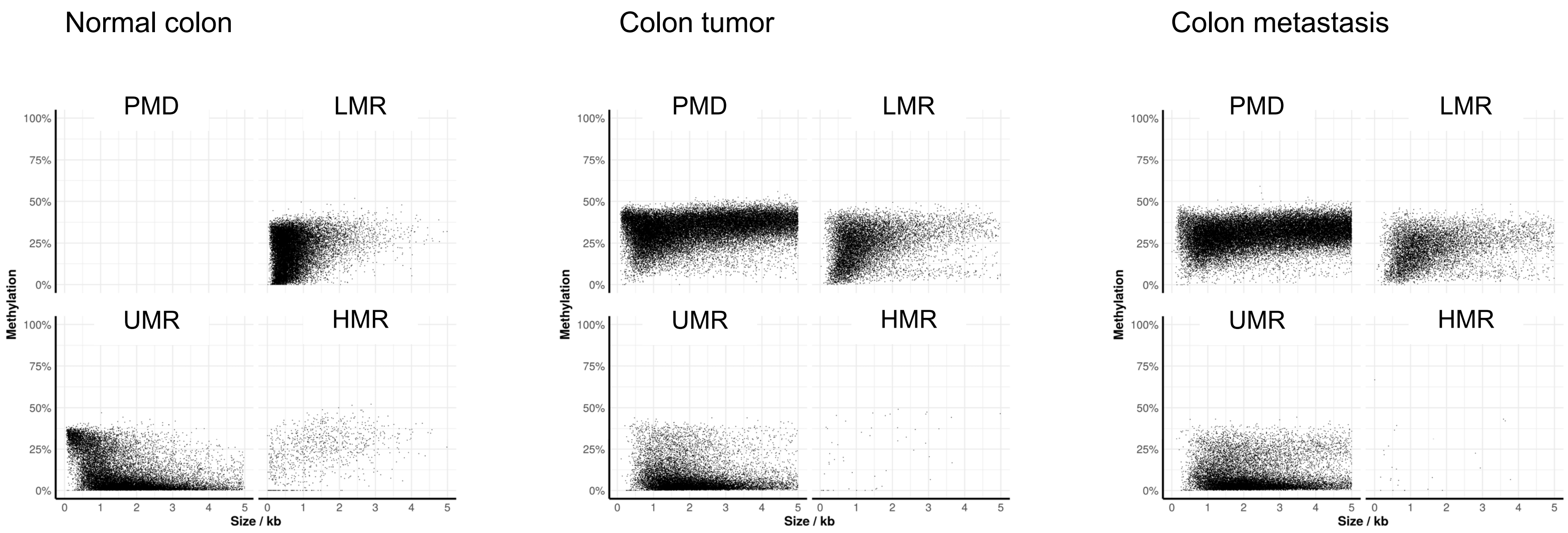

Normal colon
Colon tumor
Colon metastasis
PMD
LMR
UMR
HMR
Methylation
100%
75%
50%
25%
0%
0
1
2
3
4
5
Size / kb

Figure S16

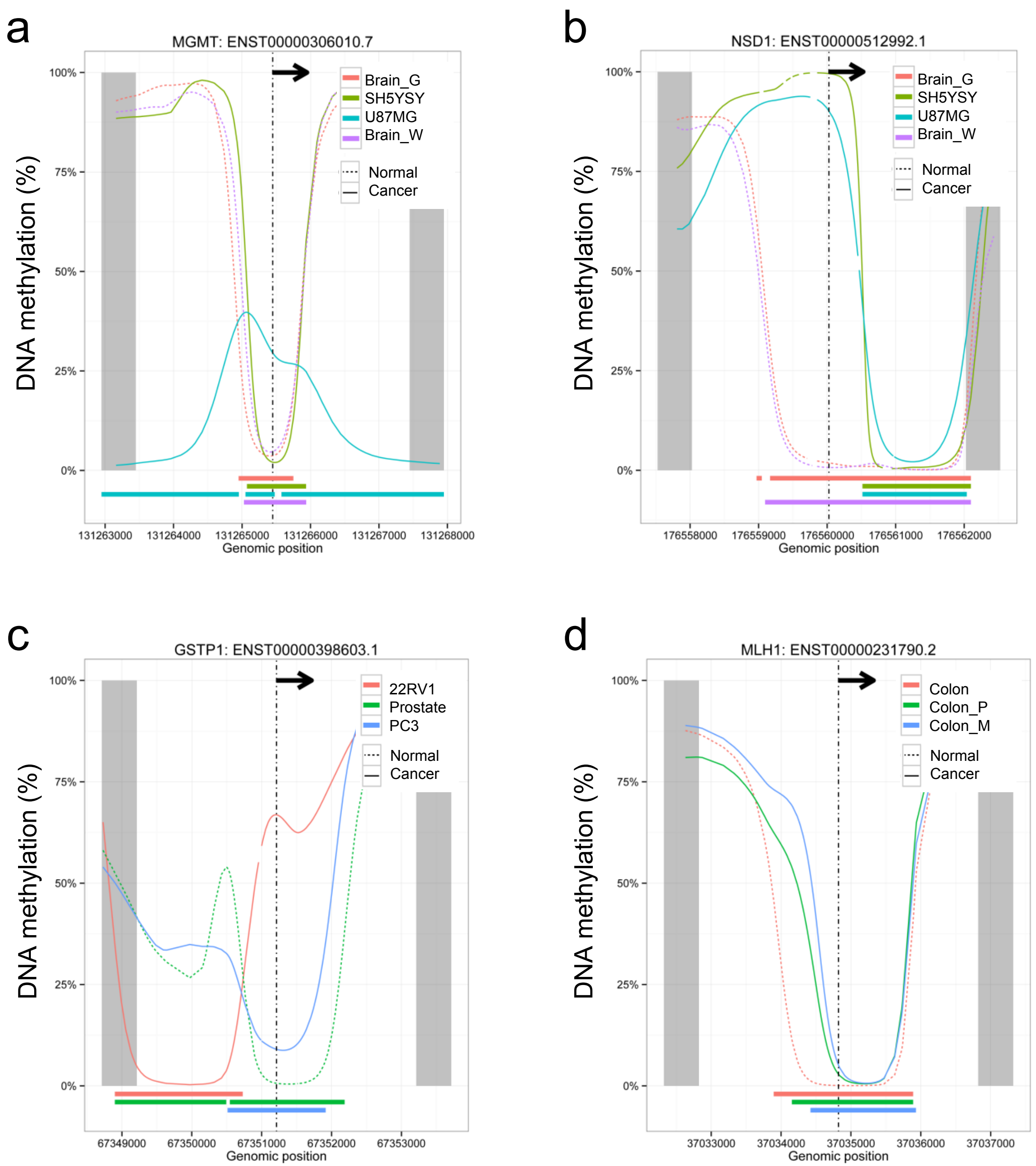
a
MGMT: ENST00000306010.7
Brain_G
SH5YSY
U87MG
Brain_W
Normal
Cancer
DNA methylation (%)
100%
75%
50%
25%
0%
131263000
131264000
131265000
131266000
131267000
131268000
Genomic position
b
NSD1: ENST00000512992.1
Brain_G
SH5YSY
U87MG
Brain_W
Normal
Cancer
DNA methylation (%)
176558000
176559000
176560000
176561000
176562000
Genomic position
c
GSTP1: ENST00000398603.1
22RV1
Prostate
PC3
Normal
Cancer
DNA methylation (%)
67349000
67350000
67351000
67352000
67353000
Genomic position
d
MLH1: ENST00000231790.2
Colon
Colon_P
Colon_M
Normal
Cancer
DNA methylation (%)
37033000
37034000
37035000
37036000
37037000
Genomic position

Figure S17

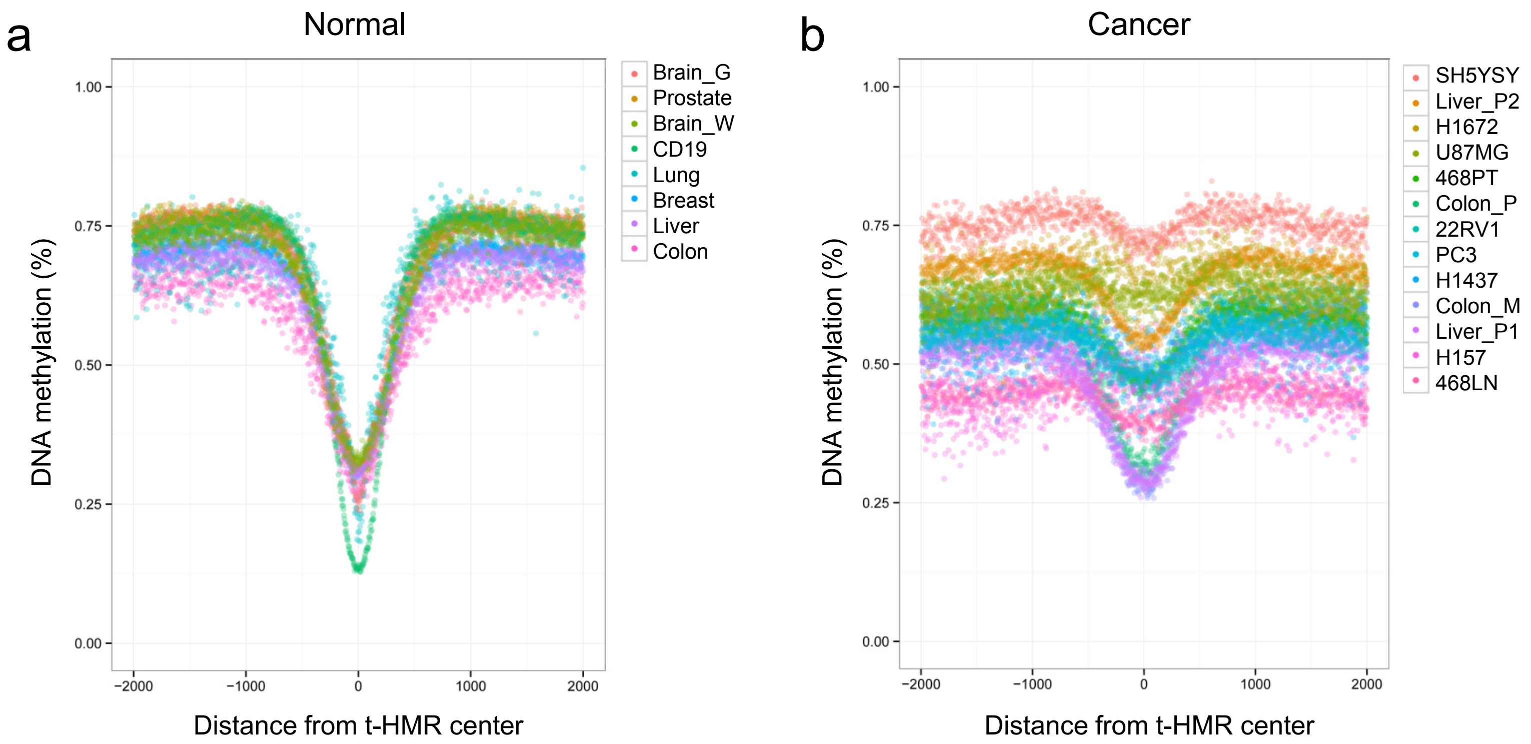
a
Normal
DNA methylation (%)
1.00
0.75
0.50
0.25
0.00
−2000
−1000
0
1000
2000
Distance from t-HMR center
Brain_G
Prostate
Brain_W
CD19
Lung
Breast
Liver
Colon
b
Cancer
DNA methylation (%)
1.00
0.75
0.50
0.25
0.00
−2000
−1000
0
1000
2000
Distance from t-HMR center
SH5YSY
Liver_P2
H1672
U87MG
468PT
Colon_P
22RV1
PC3
H1437
Colon_M
Liver_P1
H157
468LN

Table S1: Common, frequent and specific HMRs in normal human samples.

Tissue	Origin	# HMRs	Genome-wide Coverage (bp)	Genome-wide Coverage (%)	AVR HMR Size (bp)
CD19	common	4515	6256437	0,21%	927
CD19	frequent	9503	11261746	0,37%	765
CD19	specific	14092	7917316	0,26%	436
Brain	common	4209	6757362	0,22%	1107
Brain	frequent	8562	11850842	0,39%	917
Brain	specific	4932	2809802	0,09%	428
Breast	common	4818	8262050	0,27%	1251
Breast	frequent	11411	15889310	0,52%	993
Breast	specific	9797	7152316	0,24%	572
Colon	common	4265	6849339	0,23%	1171
Colon	frequent	8617	12180402	0,40%	1010
Colon	specific	3472	3814410	0,13%	823
Liver	common	4650	8366788	0,28%	1213
Liver	frequent	9924	15593961	0,51%	1051
Liver	specific	10832	14123824	0,47%	914
Lung	common	4223	6142345	0,20%	1008
Lung	frequent	8342	10471696	0,34%	831
Lung	specific	1226	609271	0,02%	352
Prostate	common	4908	8481850	0,28%	1247
Prostate	frequent	11840	16613772	0,55%	1007
Prostate	specific	11655	9905193	0,33%	717

Table S2: Top 100 most frequently hypermethylated HMRs in promoters throughout all cancer types.

gene	HMR ID	# probes	Total	Frequencies	ALL	BC	CC	CLL	GB	LAD	LC	LSQ	LYMPH	NB	PC	SCLC
					**Hypermethylation events per cancer type**											
TBX15	common_1730	26	820	0,74	28	53	102	14	27	225	193	90	12	0	21	55
NPHS1	common_26422	3	734	0,66	44	59	75	25	35	183	158	78	18	0	15	44
PDE4DIP	common_1766	2	723	0,65	35	38	73	8	15	208	203	69	18	0	20	36
PRDM14	common_12721	18	711	0,64	29	44	100	7	33	175	167	77	11	0	20	48
BARHL2	common_1496	29	668	0,60	28	45	97	22	34	156	125	77	17	0	16	51
EVX2	common_4365	32	650	0,58	35	44	80	26	24	173	99	83	15	0	19	52
HOXA9	common_10898	25	649	0,58	24	39	16	14	35	203	162	94	10	0	0	52
ISL2	common_21209	8	635	0,57	8	40	42	1	18	176	180	92	9	0	21	48
OTP	common_7939	14	632	0,57	21	44	73	17	28	193	120	55	17	0	20	44
C2orf40	common_3910	7	626	0,56	32	31	96	6	29	170	129	62	17	2	16	36
ALX3	common_1615	5	620	0,56	9	49	83	0	28	133	151	77	7	11	19	53
SIM1	common_10076	33	617	0,55	27	35	91	6	15	131	167	70	13	0	10	52
DMRTA2	common_1240	22	613	0,55	26	31	96	6	36	173	59	100	11	0	21	54
CHFR	common_19052	3	612	0,55	29	34	99	4	38	119	159	74	4	0	2	50
FOXG1	common_19748	29	609	0,55	43	37	97	25	0	173	117	65	8	0	18	26
ZIC2	common_19467	6	605	0,54	15	38	66	10	44	133	160	77	9	0	21	32
ADAM32	common_12529	7	604	0,54	43	22	29	16	40	123	205	55	17	5	10	39
TBX20	common_10990	34	598	0,54	34	44	100	8	28	161	63	66	15	18	10	51
BOLL	common_4491	8	597	0,54	38	39	99	5	34	107	162	72	6	0	5	30
NKX2-3	common_15449	14	594	0,53	33	43	99	11	13	149	70	86	15	0	22	53
DRD4	common_15983	8	591	0,53	17	24	95	3	12	130	191	76	9	0	6	28
CYP26C1	common_15355	6	589	0,53	20	31	71	2	21	120	162	73	12	5	23	49
C9orf53;CDKN2A	common_13384	1	583	0,52	10	45	80	9	9	105	183	62	3	2	21	54
GALR3	common_29147	4	576	0,52	24	48	55	1	33	130	203	43	3	1	11	24
THBS4	common_7959	7	571	0,51	34	30	96	15	7	138	131	58	17	0	23	22
RP11-542P2.1	common_7267	4	569	0,51	32	40	34	3	42	180	84	65	13	1	25	50
TRPM2	common_28678	4	562	0,51	29	24	92	18	9	184	93	52	17	0	3	41
GCM2	common_9084	10	560	0,50	42	35	99	13	36	146	60	41	14	0	24	50
WNT7B	common_29379	6	555	0,50	8	27	73	0	0	245	29	97	5	0	21	50
OTX2	common_19916	15	554	0,50	40	30	97	12	19	140	45	90	14	0	14	53
FOXE3	common_1212	6	553	0,50	43	37	27	20	35	117	197	34	9	0	14	20
ASIC3	common_11945	3	542	0,49	26	24	59	23	27	113	204	13	11	9	11	22
LYPD5	common_26726	9	538	0,48	41	40	89	8	22	163	53	73	3	1	2	43
LRRC3	common_28680	4	536	0,48	24	36	81	16	39	101	159	26	13	0	7	34
CCDC8	common_26861	5	534	0,48	26	47	100	9	27	121	89	44	14	17	18	22
DLX6	common_11414	24	529	0,48	32	37	70	9	22	118	146	53	8	2	21	11
SIX1	common_19956	4	525	0,47	21	36	66	12	21	152	76	87	6	0	25	23
TCTEX1D1	common_1385	12	524	0,47	8	49	96	1	2	113	142	32	11	0	24	46

PRICKLE2	common_5580	6	522	0,47	47	21	96	22	4	168	40	47	15	8	25	29
KCNA7	common_27020	6	521	0,47	40	25	70	22	31	108	163	22	15	0	0	25
EFCAB1	common_12592	9	516	0,46	44	22	99	24	25	100	152	18	17	0	5	10
ZNF782	common_13837	1	515	0,46	27	31	93	3	20	108	112	53	11	0	9	48
OTX2	common_19918	17	515	0,46	39	31	90	5	27	131	25	91	13	0	14	49
SCGN	common_9181	10	511	0,46	45	24	50	28	12	180	118	21	18	0	0	15
FAM19A1	common_5598	6	510	0,46	21	25	89	16	18	96	178	23	16	0	10	18
HOPX	common_6942	10	507	0,46	9	34	81	0	1	237	40	37	6	0	24	38
OXT	common_27507	3	505	0,45	15	40	50	0	35	105	149	52	2	3	22	32
PAX6	common_16321	19	504	0,45	22	23	94	3	41	98	70	76	9	0	16	52
PRAC	common_23940	10	503	0,45	23	40	25	11	19	165	73	81	11	0	1	54
TAL1	common_1209	19	497	0,45	12	43	31	1	16	124	148	44	7	0	21	50
SLC13A3	common_28033	4	497	0,45	36	25	93	15	19	160	44	41	12	0	25	27
JPH4	common_19694	2	496	0,45	22	43	93	5	25	97	83	63	15	2	9	39
NKX2-4	common_27659	14	496	0,45	21	28	90	7	21	98	146	46	8	0	9	22
MAST1	common_25864	8	493	0,44	6	39	37	0	17	66	213	33	13	19	18	32
SOX1	common_19551	48	492	0,44	37	34	94	17	18	127	53	54	14	0	15	29
DMRTA2	common_1241	32	490	0,44	26	30	59	13	3	108	119	61	8	0	19	44
USP44	common_18456	8	486	0,44	40	37	92	0	32	23	199	33	15	0	8	7
ZSCAN30	common_24874	5	485	0,44	9	14	53	4	5	113	205	52	9	2	8	11
ZNF573	common_26487	5	483	0,43	41	26	94	13	13	91	123	51	15	0	3	13
MAGI1	common_5586	2	483	0,43	47	21	32	31	0	147	115	47	18	2	5	18
VSX2	common_20093	13	482	0,43	18	27	96	1	10	132	95	38	10	0	19	36
WT1	common_16333	3	479	0,43	13	44	88	16	4	155	36	64	14	0	21	24
KIF19	common_24295	8	479	0,43	10	37	75	10	18	122	111	48	8	1	20	19
UCHL1	common_6850	8	478	0,43	44	23	82	3	1	95	173	20	12	0	24	1
TMC4	common_27250	5	476	0,43	12	45	47	3	26	73	134	55	9	10	18	44
CHD5	common_216	3	475	0,43	38	29	9	34	33	96	167	20	10	12	1	26
ZNF814	common_27390	9	475	0,43	18	15	81	0	2	109	138	68	8	0	3	33
MLNR	common_19313	5	474	0,43	24	20	85	19	39	70	181	9	8	0	6	13
RYR2	common_2925	10	473	0,43	43	34	103	16	22	91	67	49	14	0	6	28
KCNIP4	common_6776	7	472	0,42	46	30	96	28	5	134	53	37	15	0	2	26
TCF24	common_12705	12	470	0,42	12	25	93	0	21	79	163	42	3	0	14	18
B3GNT6	common_17055	1	470	0,42	17	13	34	4	12	183	101	41	1	0	18	46
HOXA9;RP1-170O1	common_10900	31	469	0,42	18	24	4	10	24	155	154	32	11	0	0	37
FBLL1	common_8643	7	468	0,42	42	5	85	13	8	118	158	16	8	0	7	8
ANKMY1;DUSP28	common_4920	7	467	0,42	22	4	82	6	3	72	189	37	16	0	3	33
TAC1	common_11423	13	464	0,42	42	31	81	11	28	97	39	53	13	2	18	49
SCRT1	common_13210	4	464	0,42	31	28	70	10	22	108	143	25	13	5	0	9
ACRBP	common_17677	5	464	0,42	16	28	8	1	20	74	181	57	16	12	21	30
ASPG	common_20454	5	464	0,42	44	25	89	28	19	138	15	37	16	3	6	44
IDUA	common_6506	9	462	0,42	35	32	42	2	36	72	194	29	7	0	5	8
KCNA6	common_17638	7	461	0,41	24	32	94	0	0	163	83	40	10	0	5	10
LHX1	common_23557	9	460	0,41	13	24	88	4	14	124	54	65	11	3	9	51
NKX6-2	common_15897	45	458	0,41	38	20	64	6	7	85	193	19	12	0	0	14

VSX1	common_27708	20	457	0,41	37	34	103	6	11	104	68	47	10	0	6	31
PLAC9	common_15257	6	455	0,41	32	45	69	23	12	89	68	59	17	0	21	20
PRDM15	common_28601	1	454	0,41	9	49	53	0	1	104	148	36	15	0	20	19
AKR1E2	common_14646	7	452	0,41	21	32	56	16	38	37	177	34	6	1	11	23
EPS8L2	common_15988	2	451	0,41	0	27	46	0	2	116	204	39	0	0	11	6
VAX1	common_15700	25	450	0,40	42	20	90	19	19	97	59	52	13	0	8	31
C1orf114	common_2289	9	448	0,40	24	49	96	1	0	83	77	64	2	0	24	28
GBX2	common_4853	7	447	0,40	30	29	42	7	35	95	84	64	13	1	20	27
DNAJC6	common_1375	11	446	0,40	16	34	65	1	0	160	81	28	16	0	24	21
FAM181B	common_17080	9	444	0,40	40	26	60	13	0	110	138	12	14	0	15	16
C1QL3	common_14748	7	442	0,40	22	17	87	0	10	98	183	13	2	0	1	9
TJP1	common_20618	6	440	0,40	45	24	67	31	1	130	67	29	18	0	10	18
ZIC1	common_6079	3	440	0,40	27	20	97	5	6	66	131	40	11	0	1	36
PRDM13	common_10059	25	439	0,39	40	31	80	21	18	103	20	54	13	0	11	48
ZAR1	common_6890	10	439	0,39	28	19	24	1	16	84	198	11	14	11	21	12
KCNS2	common_12868	7	438	0,39	40	9	59	11	12	102	180	7	14	0	0	4
SORCS1	common_15626	8	438	0,39	35	11	99	14	4	122	47	55	13	0	14	24

Table S3: Hypermethylated HMRs in promoters with a DNA methylation intensity >50%.

					Hypermethylation events per cancer type									
**gene**	**HMR ID**	**Nprobes**	**Intensity**	**Frequency**	**BC**	**CC**	**GB**	**LAD**	**LC**	**LSQ**	**LYMPH**	**NB**	**PC**	**SCLC**
DCDC2	common_9165	3	0,66	0,20	1	2	0	0	198	0	0	0	0	0
CTSZ	common_28216	9	0,63	0,06	0	1	17	3	1	2	0	17	0	17
PISD	common_29034	8	0,62	0,16	0	1	0	7	142	5	0	0	0	4
ADHFE1	common_12695	9	0,62	0,22	4	103	1	52	1	21	11	0	3	23
IQSEC1	common_5080	11	0,62	0,05	1	0	8	0	0	3	8	1	0	31
CDKN2A	common_13386	2	0,60	0,27	4	50	0	22	160	30	3	0	2	1
ERGIC1	common_8693	4	0,59	0,01	2	0	2	2	3	1	0	0	0	1
BCAT2	common_27005	3	0,59	0,02	0	0	9	2	0	1	2	0	7	0
TRIM13	common_19324	7	0,59	0,05	0	0	0	0	46	0	0	0	0	2
GNMT	common_9758	26	0,58	0,03	0	0	7	2	0	0	16	0	0	3
DCDC2;KAAG1	common_9164	9	0,58	0,33	7	17	44	18	176	30	7	12	3	22
SNED1	common_4937	10	0,57	0,01	0	0	8	0	0	0	6	0	0	1
SNX31	common_12887	8	0,57	0,28	12	12	6	35	194	8	13	0	3	4
SRF	common_9768	4	0,57	0,17	8	0	0	7	143	7	1	0	0	3
GPER	common_10602	4	0,57	0,06	0	18	6	3	1	5	0	0	0	27
SH3BGRL3	common_697	7	0,57	0,03	0	0	0	0	30	0	0	0	0	1
UNKL	common_21660	3	0,56	0,18	0	0	1	6	171	2	0	0	0	1
GRHL2	common_12906	4	0,56	0,26	0	1	43	1	196	0	10	4	1	4
DDAH2	common_9505	36	0,56	0,01	0	0	0	1	0	5	1	0	0	5
ARTN	common_1124	2	0,56	0,06	6	3	6	2	6	0	17	13	0	5
FAM149A	common_7490	12	0,56	0,01	2	3	0	4	0	0	4	0	0	2
IGDCC3	common_21015	6	0,56	0,04	0	34	0	5	0	0	5	0	0	0
NCOA4	common_15020	9	0,56	0,06	5	3	3	2	0	8	15	0	12	10
ZNF788	common_25823	6	0,56	0,32	1	84	3	26	145	28	6	11	4	16
EDEM2	common_27820	6	0,56	0,11	3	7	0	6	79	14	0	0	0	1
SEPT9	common_24422	13	0,56	0,30	2	71	0	19	173	24	0	0	14	1
ARAP3	common_8433	8	0,56	0,03	1	0	10	1	3	0	7	8	0	3
AMPH	common_11011	8	0,56	0,17	1	99	1	35	26	2	6	0	3	0

FAM109B	common_29283	3	0,56	0,26	2	2	12	11	196	15	4	7	0	16
DCLK1	common_19232	8	0,55	0,14	0	96	0	5	23	0	14	0	0	0
AS3MT	common_15582	8	0,55	0,05	4	25	0	3	2	2	12	0	0	2
FIGN	common_4253	10	0,55	0,12	3	95	0	8	0	0	17	0	0	2
BCAT2	common_27004	3	0,55	0,03	0	0	0	0	27	0	0	0	0	0
THEM4	common_1942	8	0,55	0,04	2	1	11	1	0	15	0	0	0	13
TJP3	common_25468	3	0,55	0,13	0	0	0	1	132	0	0	0	0	0
USP44	common_18456	8	0,55	0,44	37	92	32	23	199	33	15	0	8	7
FDFT1	common_12233	5	0,55	0,02	0	1	0	0	0	4	14	0	0	1
C8orf42	common_12104	6	0,55	0,13	3	89	4	4	9	3	14	0	0	6
ZSCAN30	common_24874	5	0,55	0,47	14	53	5	113	205	52	9	2	8	11
TRAF3IP2	common_10157	8	0,54	0,04	0	0	6	0	9	0	2	19	0	8
ANO5	common_16287	10	0,54	0,10	3	71	0	10	2	3	11	0	1	0
PPFIA3	common_27026	5	0,54	0,32	18	57	0	30	139	56	10	0	0	11
C20orf194	common_27518	13	0,54	0,05	0	44	0	1	7	0	0	0	0	0
TPST1	common_11195	11	0,54	0,01	0	12	0	0	0	0	0	0	0	0
ACSL6	common_8162	9	0,54	0,05	0	24	0	5	10	0	8	0	0	1
HFE	common_9189	4	0,54	0,09	11	0	6	9	2	3	7	19	17	21
RASL11B	common_6907	4	0,54	0,26	0	22	13	28	192	4	1	0	0	1
MYO15A	common_23284	3	0,54	0,19	2	3	7	4	135	15	12	0	15	3
C8orf86	common_12517	3	0,54	0,01	0	1	0	0	0	2	3	0	0	5
GPR126	common_10329	7	0,54	0,02	5	0	4	1	0	0	10	0	0	1
SUOX	common_18209	11	0,54	0,02	0	0	0	0	0	1	15	0	0	0
ZNF790	common_26463	14	0,54	0,06	0	48	0	5	0	5	1	0	0	0
TTC12	common_17234	11	0,54	0,03	0	0	14	0	0	3	3	0	0	6
ZNF783	common_11896	5	0,54	0,22	6	2	0	6	199	10	3	0	0	0
EVL	common_20349	9	0,54	0,11	1	90	0	4	13	0	0	0	0	1
EVC2;EVC	common_6632	11	0,54	0,11	1	90	2	2	12	0	3	0	0	4
C1orf216	common_931	6	0,53	0,01	0	4	1	0	0	0	0	0	0	6
STAT5A	common_23713	2	0,53	0,02	0	0	5	4	0	2	0	0	1	5
RASGRF1	common_21257	22	0,53	0,12	4	85	1	7	17	0	1	0	0	3
ZNF793	common_26480	13	0,53	0,12	1	85	0	14	19	4	1	0	0	2
ERBB2	common_23634	10	0,53	0,06	0	1	8	0	8	1	16	4	0	27

EIF4EBP3	common_8320	12	0,53	0,03	0	0	0	9	0	7	12	0	0	6
MYO15A	common_23285	2	0,53	0,05	0	0	7	2	18	1	0	12	0	9
CCDC149	common_6786	4	0,53	0,02	0	2	0	0	6	0	13	0	0	2
MR1	common_2376	3	0,53	0,06	0	9	5	2	3	3	0	12	0	24
MSMP	common_13481	2	0,53	0,04	1	0	0	2	0	28	0	0	5	0
SLC16A12	common_15323	14	0,53	0,19	13	94	8	26	13	5	12	0	12	4
ECHDC2	common_1270	4	0,53	0,02	0	1	8	2	0	2	0	0	0	3
CCNJ	common_15384	14	0,53	0,16	0	0	0	0	159	4	0	0	0	0
MARCH2	common_2720	14	0,53	0,03	0	1	5	3	0	6	16	0	0	4
CELF6	common_21109	9	0,53	0,19	0	8	0	2	180	0	0	0	1	0
RBMS1	common_4232	6	0,53	0,18	0	42	1	0	134	0	0	0	2	1
SYNM	common_21510	11	0,53	0,18	3	19	11	15	119	2	8	0	0	0
GREB1L	common_24799	14	0,53	0,13	0	77	24	8	0	5	12	0	0	1
CRYM	common_21980	3	0,53	0,38	36	32	34	40	169	27	16	0	7	20
BHLHA15	common_11432	3	0,53	0,24	9	9	8	6	171	8	14	0	15	2
FAM20A	common_24246	6	0,53	0,09	10	66	5	0	0	0	5	0	1	1
DIXDC1	common_17223	8	0,53	0,10	0	10	0	24	46	2	13	0	0	4
PTPRG	common_5558	17	0,53	0,01	1	2	0	3	0	0	5	0	0	0
SPATA32	common_23838	11	0,53	0,36	4	90	0	47	142	39	7	0	2	34
ZNF148	common_5844	5	0,53	0,35	11	90	4	22	169	22	14	0	0	25
VEPH1	common_6145	11	0,53	0,05	0	0	12	1	0	0	7	13	0	17
NPAS2	common_3867	12	0,53	0,01	0	0	0	1	0	0	5	0	0	5
STOM	common_14040	3	0,53	0,09	0	69	0	3	0	10	0	0	0	9
FAM65A	common_22454	5	0,53	0,10	1	21	2	5	66	5	0	0	1	4
CLHC1	common_3443	4	0,53	0,09	0	9	27	20	12	9	0	0	6	11
NRG1	common_12473	10	0,53	0,10	2	80	0	8	1	0	5	0	3	2
TMEM88B	common_65	2	0,52	0,04	0	22	0	8	9	1	4	0	0	1
FAM126A	common_10841	13	0,52	0,03	7	18	0	1	0	0	0	0	0	1
CHST1	common_16425	10	0,52	0,04	0	33	0	5	0	0	1	0	0	0
OXT	common_27507	3	0,52	0,49	40	50	35	105	149	52	2	3	22	32
ZNF264	common_27359	14	0,52	0,05	1	46	0	1	2	3	0	0	0	0
KCNK5	common_9693	9	0,52	0,03	5	0	12	0	3	1	0	0	0	5
PHF19	common_14028	4	0,52	0,15	0	0	41	2	69	7	0	2	0	27

DRD4	common_15983	8	0,52	0,57	24	95	12	130	191	76	9	0	6	28
MPZ	common_2193	2	0,52	0,31	46	51	1	39	140	20	5	0	7	6
FBXO10	common_13518	2	0,52	0,07	1	44	0	9	2	5	7	0	0	2
LRRFIP1	common_4873	11	0,52	0,10	0	76	1	12	0	2	5	0	0	0
EPM2AIP1;MLH1	common_5214	39	0,52	0,02	0	16	0	0	0	1	0	0	0	2
EPB49	common_12311	2	0,52	0,02	0	1	10	2	2	0	0	3	0	3
APBB1	common_16121	19	0,52	0,02	0	10	0	1	9	0	0	0	0	0
AQP5	common_18003	4	0,52	0,40	16	93	29	50	165	8	8	1	25	6
FNBP1	common_14287	8	0,52	0,01	0	15	0	0	0	0	0	0	0	0
MYO5C	common_20893	8	0,52	0,02	0	0	0	0	1	1	12	0	0	2
TNFAIP8	common_8114	8	0,52	0,10	6	29	1	18	2	13	2	0	5	23
ANKEF1	common_27593	9	0,52	0,01	0	1	7	0	0	0	6	0	0	0
LONRF2	common_3863	7	0,52	0,12	1	99	0	2	11	1	2	0	0	1
DAB1	common_1316	15	0,52	0,15	2	95	0	27	11	0	12	0	0	2
RUNDC3B	common_11362	11	0,52	0,06	0	12	23	9	0	3	7	0	0	4
H2AFJ	common_17790	15	0,52	0,06	0	10	12	5	4	12	0	0	0	20
SH3RF2	common_8469	8	0,52	0,04	1	1	2	0	1	0	1	12	0	23
C1QL1	common_23820	4	0,52	0,36	7	72	0	56	193	9	2	3	11	6
CYP26B1	common_3609	2	0,52	0,23	30	4	13	25	111	29	4	1	7	11
SLC2A2	common_6207	5	0,52	0,21	46	15	13	53	6	35	0	2	21	24
C9orf3	common_13811	3	0,52	0,01	0	0	5	0	5	0	2	1	0	1
SASH1	common_10361	14	0,52	0,01	1	1	0	0	0	0	8	0	0	3
ANKRD6	common_10020	5	0,52	0,04	0	34	0	0	0	1	2	0	0	0
SCNN1A	common_17663	3	0,52	0,02	0	1	4	1	0	0	2	0	0	11
ANKRD28	common_5130	2	0,52	0,09	1	12	0	0	30	1	0	4	5	35
ALS2CL	common_5329	9	0,52	0,02	0	1	5	0	1	1	2	0	0	13
NAV2	common_16275	5	0,52	0,02	0	1	8	0	2	1	2	1	0	2
RNF180	common_7833	7	0,52	0,13	9	63	0	31	8	2	17	0	0	5
DNAH11	common_10825	9	0,52	0,11	3	85	0	4	3	0	12	0	0	1
METTL24	common_10147	6	0,52	0,32	16	90	17	40	113	15	16	0	5	7
PDZD2	common_7677	6	0,52	0,09	0	68	2	1	8	0	11	0	2	1
SMTN	common_29025	4	0,52	0,22	10	42	4	44	74	7	0	2	12	27
THRB	common_5159	26	0,52	0,04	1	3	6	8	0	0	17	0	0	6

PPAP2B;PRKAA2	common_1314	15	0,52	0,09	0	33	0	5	30	0	17	0	0	1
OSBPL1A	common_24830	7	0,52	0,02	0	3	0	0	0	0	13	0	0	9
UGGT2	common_19432	12	0,52	0,01	0	0	0	0	0	1	13	0	0	1
CYGB;PRCD	common_24398	10	0,52	0,22	9	31	0	32	130	9	8	0	0	4
ENTPD3	common_5252	7	0,52	0,25	4	29	25	3	175	0	9	2	1	4
AGBL2	common_16475	6	0,51	0,01	0	2	2	3	0	1	0	2	0	3
CLUAP1	common_21786	13	0,51	0,02	0	1	0	2	0	22	0	0	0	0
IQCJ-SCHIP1	common_6160	2	0,51	0,08	2	2	12	18	0	3	3	4	0	32
EFNB2	common_19496	10	0,51	0,18	0	1	3	2	177	1	1	0	0	0
STAT6	common_18249	2	0,51	0,04	1	0	23	0	2	1	0	6	1	8
PHLDB1	common_17303	8	0,51	0,01	0	1	5	0	2	0	0	3	0	3
C3orf80	common_6165	13	0,51	0,03	0	16	0	1	11	0	0	0	0	0
CYP1B1	common_3318	27	0,51	0,02	0	3	2	2	0	2	2	0	0	5
EHD1	common_16713	3	0,51	0,03	4	1	6	8	0	3	0	0	0	9
CPAMD8	common_26041	8	0,51	0,10	10	62	4	6	7	0	4	0	0	4
SH3GL3	common_21312	11	0,51	0,13	1	78	7	24	13	1	3	0	0	6
FOXE3	common_1212	6	0,51	0,49	37	27	35	117	197	34	9	0	14	20
CAST	common_8044	12	0,51	0,05	3	1	0	15	0	5	15	0	6	4
TM9SF1;CHMP4A;MDP1	common_19715	6	0,51	0,10	3	0	0	1	94	0	0	0	3	0
WWTR1	common_6093	17	0,51	0,01	0	7	5	0	0	0	0	0	0	3
RBP1	common_6020	12	0,51	0,22	3	85	7	11	84	4	8	0	20	0
SLC34A2	common_6794	7	0,51	0,19	17	23	38	4	40	30	15	0	18	8
RARRES1;MFSD1	common_6156	6	0,51	0,07	2	2	4	6	6	17	9	1	14	11
DOCK1	common_15824	8	0,51	0,02	0	1	0	2	2	3	7	0	0	2
ABCA3	common_21707	5	0,51	0,01	0	0	0	0	12	0	0	0	0	0
RERG	common_17792	8	0,51	0,09	0	59	0	2	11	6	5	0	1	3
RBM23	common_19669	10	0,51	0,01	0	0	0	1	12	0	0	0	0	0
BOLL	common_4491	8	0,51	0,55	39	99	34	107	162	72	6	0	5	30
ZFP64	common_28127	9	0,51	0,39	19	89	8	26	194	2	7	0	24	20
TTC28	common_28965	4	0,51	0,36	27	30	1	112	60	53	9	4	23	43
MGST2	common_7257	8	0,51	0,02	1	0	5	1	0	1	2	6	0	4
NGF	common_1697	10	0,51	0,19	14	97	3	17	35	1	12	0	6	5
TIMP3	common_29049	4	0,51	0,06	3	21	1	10	3	3	5	3	0	13

MME	common_6123	16	0,51	0,10	12	56	3	5	5	1	8	0	1	7
LPHN2	common_1440	20	0,51	0,05	2	38	0	4	0	0	4	0	0	1
TENM3	common_7440	9	0,51	0,10	0	64	0	9	13	0	12	0	0	0
ZNF85	common_26222	8	0,51	0,15	0	81	1	18	20	25	2	0	0	2
SPINT2	common_26502	10	0,51	0,20	0	1	18	0	177	0	2	0	0	1
STARD13	common_19220	9	0,51	0,02	0	1	0	0	1	0	5	1	0	8
CELF2	common_14692	13	0,51	0,18	24	3	0	9	133	3	4	0	3	5
CDKL2	common_6989	11	0,51	0,28	36	5	17	5	200	6	1	0	7	2
ZNF461	common_26458	12	0,51	0,06	0	42	2	4	2	6	0	0	0	0
XRCC5	common_4610	3	0,51	0,01	2	4	2	1	0	3	0	0	0	0
SDC2	common_12855	15	0,51	0,10	1	86	0	9	0	0	5	0	0	1
CXCL2	common_6981	12	0,51	0,03	3	1	5	0	0	3	15	0	3	1
FAM174B	common_21445	8	0,51	0,08	0	2	17	1	61	0	1	0	0	0
TLR3	common_7488	5	0,51	0,03	0	0	0	1	1	0	0	17	0	12
ZNF829;ZNF568	common_26465	14	0,51	0,15	0	83	0	21	19	22	1	0	0	5
EFHA2	common_12269	8	0,51	0,12	8	90	0	6	2	0	12	0	0	1
GNAL	common_24749	10	0,51	0,03	3	10	6	1	4	0	2	0	0	2
C1orf114	common_2289	9	0,51	0,42	49	96	0	83	77	64	2	0	24	28
ROR1	common_1364	12	0,51	0,01	1	1	0	0	8	0	5	0	0	0
PLIN2	common_13359	2	0,51	0,03	0	7	2	2	2	11	0	0	0	11
CMAHP	common_9178	5	0,51	0,06	5	7	0	7	10	2	0	3	3	20
LYNX1	common_13127	14	0,51	0,11	4	77	0	12	6	5	0	0	1	4
RP11-1102P16.1	common_12728	5	0,51	0,15	3	63	5	22	29	2	14	0	6	4
RIMS1	common_9922	9	0,51	0,11	1	81	0	7	15	1	3	0	0	1
FAM159A	common_1264	5	0,51	0,29	16	73	12	27	140	16	2	0	2	3
GABPB2	common_1915	12	0,51	0,01	0	1	0	1	0	2	0	2	0	5
SFTPD	common_15253	3	0,51	0,02	0	15	1	0	0	0	0	0	0	0
EFCAB12	common_5932	8	0,51	0,02	0	0	0	2	5	1	14	0	0	1
OBSL1	common_4712	6	0,51	0,31	17	67	4	48	165	4	6	0	0	2
RNF217	common_10212	13	0,51	0,11	8	60	4	11	1	2	17	0	1	2
KRT8	common_18073	5	0,51	0,02	0	1	12	0	0	0	0	2	0	2
BCL2L2;BCL2L2-PABPN1	common_19685	5	0,50	0,02	0	0	0	0	0	0	16	0	0	1
ADAM32	common_12529	7	0,50	0,54	22	29	40	123	205	55	17	5	10	39

ZC3H13	common_19295	15	0,50	0,02	0	0	0	0	20	0	0	0	0	0
KCNS2	common_12868	7	0,50	0,38	9	59	12	102	180	7	14	0	0	4
ZAR1	common_6890	10	0,50	0,41	19	24	16	84	198	11	14	11	21	12
ATRAID;CAD	common_3231	5	0,50	0,03	0	1	2	0	27	0	0	0	1	0
VRK2	common_3458	10	0,50	0,04	0	0	33	0	1	0	0	6	0	1
HOOK1	common_1332	12	0,50	0,03	0	0	21	0	0	0	10	0	0	1
PCDP1	common_4020	6	0,50	0,02	1	6	0	0	0	0	11	0	0	1
FAM196A	common_15826	11	0,50	0,30	0	77	0	38	168	4	10	0	0	2
ZNF132	common_27416	10	0,50	0,28	24	97	0	36	56	54	3	0	0	12
ZNF483	common_13936	2	0,50	0,35	14	66	2	32	196	29	12	0	3	2
ARL10	common_8763	10	0,50	0,08	10	28	0	19	17	4	0	0	0	1
TNIP3	common_7204	2	0,50	0,22	14	32	1	28	45	67	16	0	4	18
SORCS2	common_6678	8	0,50	0,10	6	85	1	1	0	0	5	0	0	1
CADPS2	common_11674	17	0,50	0,01	0	0	0	0	1	0	10	0	0	0
HTRA1	common_15772	15	0,50	0,02	0	12	0	1	0	0	2	0	0	2
PIK3R1	common_7853	9	0,50	0,03	0	11	0	2	0	12	0	0	0	7
RHOF	common_18836	8	0,50	0,02	0	0	1	1	0	6	0	0	0	16
ACP5	common_25803	7	0,50	0,31	9	48	34	14	145	18	2	11	23	7
NUMBL	common_26611	10	0,50	0,07	0	0	0	0	68	0	0	0	0	0
CRH	common_12693	7	0,50	0,05	0	49	0	0	0	3	3	0	0	0
GPC6	common_19414	13	0,50	0,11	6	90	0	6	0	3	6	0	0	0
PAQR9	common_6051	8	0,50	0,15	16	80	2	44	0	7	2	0	0	0
HENMT1	common_1578	4	0,50	0,30	8	17	40	28	185	12	0	0	2	10
ANKRD24	common_25494	2	0,50	0,16	4	24	2	26	92	1	8	0	0	0
FN3K	common_24652	5	0,50	0,02	0	2	0	4	0	2	10	0	0	1
DPYSL3	common_8483	9	0,50	0,14	11	86	0	8	26	1	13	0	0	1
SEMA7A	common_21150	13	0,50	0,05	1	12	0	1	37	0	0	0	0	0
CELF4	common_24890	2	0,50	0,26	6	63	10	8	145	5	10	6	0	10
FKBP5	common_9629	6	0,50	0,01	0	1	6	3	0	0	0	0	0	4
FAM19A4	common_5599	12	0,50	0,22	8	94	15	41	35	10	10	0	0	11
MTHFD2	common_3644	12	0,50	0,18	0	0	0	0	182	0	0	0	0	0
BSX	common_17389	7	0,50	0,11	4	87	1	14	5	2	2	0	0	0
SYT9	common_16132	11	0,50	0,28	4	95	1	94	40	7	15	0	3	19

DPP10	common_3995	12	0,50	0,24	13	98	28	26	39	14	12	0	9	4
PXDC1	common_9003	9	0,50	0,02	1	17	0	3	0	1	2	0	0	0
EXOSC6	common_22514	8	0,50	0,01	0	0	0	8	1	1	0	0	0	1
P2RY2	common_16987	12	0,50	0,02	0	0	7	0	2	1	5	0	0	4
PHACTR1	common_9097	5	0,50	0,03	0	1	0	0	0	0	0	15	5	5
